# Paf1C regulates the *Neurospora* circadian clock by promoting the transcription elongation of *frequency*

**DOI:** 10.1371/journal.pgen.1011926

**Published:** 2025-10-23

**Authors:** Mengmeng Zhou, Yunpeng Zhao, Yubo He, Zeyu Duan, Xiao Liu, Qun He

**Affiliations:** 1 MOA Key Laboratory of Soil Microbiology, College of Biological Sciences, China Agricultural University, Beijing, China; 2 Department of Physiology, The University of Texas Southwestern Medical Center, Dallas, Texas, United States of America; 3 State Key Laboratory of Microbial Diversity and Innovative Utilization, Institute of Microbiology, Chinese Academy of Sciences, Beijing, China; 4 College of Life Sciences, University of Chinese Academy of Sciences, Beijing, China; Charité - Universitätsmedizin Berlin, GERMANY

## Abstract

The circadian rhythm is crucial for organisms to adapt timely to external environmental changes, and the operation of the circadian oscillator relies on the precise transcriptional regulation of clock genes, a process that is highly conserved across species. The activation and repression of transcriptional initiation of clock genes have been extensively studied. However, the regulation of transcriptional elongation remains largely unexplored. Here, we showed that the RNA Polymerase II Associated Factor 1 complex (Paf1C) is required for maintaining the normal circadian rhythm in *Neurospora*. The loss of PAF-1, CTR-9 or RTF-1 subunit of Paf1C led to a shorter circadian period and advanced phase. Mechanistically, the PAF-1 and CTR-9 subunits promote the transcription of the clock gene *frequency* (*frq*) by enhancing the enrichment of not only histone H2B ubiquitination (H2BK131ub), but also the phosphorylation of Ser2 in RNA Polymerase II CTD and H3K36 trimethylation (H3K36me3) at the *frq* ORF region. Moreover, the other subunit RTF-1 promotes *frq* transcription by controlling global H2BK131ub through interaction with the RAD-6/BRE-1 ubiquitin conjugase-ligase complex. Surprisingly, a highly conserved region within RTF-1 nearly rescues global H2BK131ub and the circadian clock defects in *rtf-1*^*KO*^ strains. Taken together, these results indicate that Paf1C regulates the *Neurospora* circadian clock by promoting histone H2B ubiquitination and facilitating transcription elongation of the *frq* gene.

## Author summary

While the transcriptional regulation of clock genes is fundamental to circadian clock function, research has largely focused on their activation and repression of transcription initiation, leaving the role of transcription elongation poorly understood. Here, we demonstrate that the RNA Polymerase II-Associated Factor 1 Complex (Paf1C) is essential for maintaining a normal circadian period in *Neurospora crassa* by promoting the transcription elongation of the core clock gene *frequency*. Mechanistically, PAF-1 and CTR-9, which form the core subunits of Paf1C, facilitate RNAP II occupancy and SET2-mediated H3K36 trimethylation and the RTF-1 subunit—despite its lower binding affinity to other Paf1C components—promotes RAD6/BRE1-mediated H2B monoubiquitination at the *frequency* locus. Together, these Paf1C-dependent chromatin modifications ensure the robustness of the circadian rhythm.

## Introduction

Circadian rhythms, functioning as self-sustaining timekeepers, are found in mammals, plants, insects, fungi and cyanobacteria [1–3]. The functioning of circadian oscillators, composed of positive and negative elements, relies on autoregulatory transcription/translation feedback loops [4,5]. In *Neurospora*, *Drosophila*, and mammals, the positive elements containing the PAS (PER-ARNT-SIM) domain serve as specific transcription activators that rhythmically activate the transcription of negative elements. On the contrary, the negative elements rhythmically inhibit the transcriptional activity of positive elements [6–8].

In the filamentous fungus *Neurospora crassa*, WHITE COLLAR 1 (WC-1) and WHITE COLLAR 2 (WC-2) form a heterodimeric complex (WCC), which rhythmically binds to the Clock (C)-box in the promoter of the *frequency* (*frq*) gene to activate its transcription [9–11]. On the other hand, FREQUENCY (FRQ) and a FRQ-interacting RNA helicase, FRH, form the FRQ-FRH complex (FFC) to suppress WCC transcription activity by promoting the CKI/II-mediated WCC phosphorylation, which shuts down *frq* transcription [12–14]. Once synthesized, FRQ is progressively phosphorylated by CKI, CKII and other kinases before its degradation through the ubiquitin-proteasome pathway. The turnover of FRQ results in derepressing the WCC, which reinitiates *frq* transcription [15–18]. Interestingly, besides the protein-based feedback loop, *frq* transcription is also regulated by its long non-coding antisense RNA, *qrf*, which is expressed rhythmically and plays an pivotal role in regulating *frq* expression and circadian output in *Neurospora* [19–21].

Similar to circadian clocks in other eukaryotes, the *Neurospora* circadian system also exhibits temperature compensation which maintains period length within a relatively constant range [22,23]. Previous studies have shown that this property is partially mediated by FRQ phosphorylation and stability, which are closely related to the circadian period length [24–26]. However, recent studies indicated that the rates of FRQ degradation are uncoupled from the circadian clock period, but the formation of the FRQ-CKI complex is the most critical process in circadian period determination. More interestingly, the temperature-compensated FRQ-CK1 interaction is a main determinant for *Neurospora* clock temperature compensation [27–29].

Epigenetic factors have been implicated in regulating gene transcription, with some participating in the function of the circadian clock [3,30–32]. In *Neurospora,* a recent study shows that under nutrient-deprived conditions, such as amino acid starvation, the CPC-3/CPC-1 complex recruits the acetyltransferase GCN-5 to the *frq* promoter to facilitate histone H3 acetylation, thereby maintaining the robust functions of circadian clock [33]. Upon DNA damage, the CHK1/2 checkpoint kinase–mediated phosphorylation of H3T11 within the *frq* promoter facilitates local H3K56 acetylation, which counteracts deposition of the histone variant H2A.Z and establishes a chromatin environment that supports robust circadian rhythms [34]. The chromatin remodeler IEC1-INO80 complex inhibits *frq* transcription by reassembling the suppressive environment at the *frq* promoter [35]. The methyltransferase SET-2 prevents WCC-independent *frq* transcription by maintaining proper levels of histone H3K36 methylation and H3 acetylation at the *frq* locus [36]. In mammals, the E3 ligase Rnf20-mediated H2Bub at clock gene loci, *Per* and *Cry*, is required for maintaining the normal circadian period [37]. In *Arabidopsis*, HISTONE MONOUBIQUITINATION1 (HUB1) and HUB2 complex, an E3 ubiquitin ligase, interacts with RNA-binding proteins SPEN3 and KHD1 to regulate the pre-mRNA processing of the circadian clock gene, *CCA1* [38]. These results suggest that epigenetics play key roles in the oscillation of circadian clock genes in eukaryotes.

The RNA Polymerase II Associated Factor 1 complex (Paf1C), composed of five subunits-Paf1, Ctr9, Cdc73, Leo1, and Rtf1, is highly conserved across eukaryotes. Interestingly, Rtf1 has been shown to interact less tightly with other Paf1C components in higher eukaryotes and to possess some Paf1C-independent functions in certain contexts [39–41]. Structural biology studies have shown that the yeast Ctr9 and Paf1 form a heterodimer, which is necessary for assembling the complete five-subunit Paf1C [42]. In contrast, *in vitro* purified Rtf1 does not associate with the other four subunits to form Paf1C in *Myceliophthora thermophila* [43]. Functionally, Paf1C has been extensively studied for its role in regulating a subset of gene transcription by promoting the phosphorylation of RNA Polymerase II CTD at serine2 (Ser2p) and specific histone modifications [44–48]. The Ser2p usually recruits the methyltransferase SET-2 to the coding regions of actively transcribed genes to methylate histone H3 lysine 36 [49–51]. It has been reported that the loss of Paf1 or Ctr9, but not Rtf1, profoundly decreases the levels of H3K36me3 at the *PMA1* and *PYK1* loci in *S. cerevisiae* [52,53]. Paf1C is also known to facilitate histone H2Bub by promoting the recruitment of Rad6 and Bre1, which are the respective ubiquitin-conjugating enzyme E2 and the ubiquitin ligase E3, to the coding region of actively transcribed genes [54]. It’s worth noting that the Rtf1 subunit plays most important role in this progress [54–57]. Interestingly, a small region in Rtf1, named histone modification domain (HMD), restores global H2Bub in *rtf1Δ* cells in *S. cerevisiae* [58,59]. More importantly, the key amino acids required for H2Bub within the HMD are conserved across species, indicating that the mechanism by which Rtf1 regulates H2Bub is highly conserved. Taken together, these genetic and biochemical data strongly suggest functional distinctions among the subunits of Paf1C.

Although the transcriptional activation and repression of *frq* expression have been studied extensively [35,60–62], the role of transcription elongation factors in *frq* regulation is still less understood. As is well known, the Paf1C is a well-studied and highly conserved transcriptional elongation factor in eukaryotic cells. So, we decided to determine whether the loss of Paf1C subunit affected the circadian clock in *Neurospora*. In the present study, we showed that deletion of *paf-1*, *ctr-9* or *rtf-1* resulted in a shorter circadian conidiation period and advanced phases. The Paf1C rhythmically bound to the *frq* locus. Loss of PAF-1 or CTR-9 resulted in dramatically reduced levels of FRQ protein and *frq* mRNA, due to the decreased levels of phosphorylated Ser2 of RNAPII CTD, H3K36me3, and H2BK131ub at the *frq* locus. Further studies indicate that loss of RTF-1 abolishes global H2BK131ub, including at the *frq* locus. Intriguingly, overexpression of the HMD of RTF-1 rescues global H2Bub and the circadian period in the *rtf-1*^*KO*^ strain. Taken together, these findings suggest that Paf1C maintains a normal circadian period by facilitating histone H2B ubiquitination and promoting *frq* transcription elongation in *Neurospora*.

## Results

### The Paf1 complex is required for the normal period of *N. crassa* circadian rhythm

**Fig 1:**
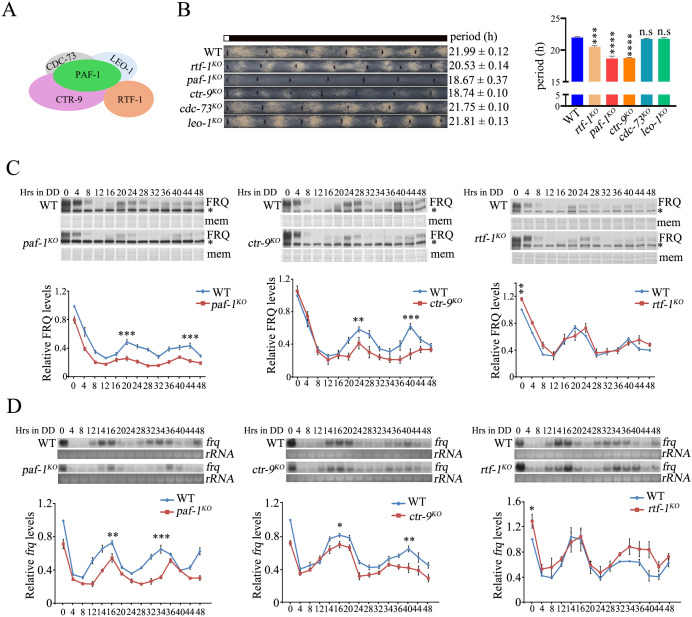
The Paf1 complex is required for the circadian rhythm of *Neurospora* by regulating rhythmic expression of *frq.* (A) Diagram showing the composition of Paf1 complex in *Neurospora crassa*. (B) Race tube assay (Left) and statistical analyses (Right) showing the circadian conidiation rhythms and period length of WT, *rtf-1*^*KO*^, *paf-1*^*KO*^, *ctr-9*^*KO*^, *cdc-73*^*KO*^, *leo-1*^*KO*^ strains. Error bars are means ± SD. (n = 3). Significance difference was assessed by Ordinary one-way ANOVA Multiple comparisons. n.s P ≥ 0.05, ****P < 0.0001. (C) Western blot analysis (Upper) and quantification (Lower) of the levels of FRQ protein in the WT, *paf-1*^*KO*^, *ctr-9*^*KO*^, *rtf-1*^*KO*^ strains. Asterisks indicate nonspecific bands. Samples were grown in DD for the indicated hours before harvest. The PVDF membrane (mem) stained with Coomassie blue was used as a loading control. Error bars are means ± SD. (n = 3). Significance difference was assessed by Student’s *t*-*t*est. *P < 0.05, **P < 0.01, ***P < 0.001. (D) Northern blot analysis (Upper) and quantification (Lower) of the levels of *frq* mRNA in the WT, *paf-1*^*KO*^, *ctr-9*^*KO*^, *rtf-1*^*KO*^ strains. The ribosome RNA (rRNA) bands stained by ethidium bromide were used as a loading control for each sample. Error bars are means ± SD. (n = 3). Significance difference was assessed by Student’s *t*-test. *P < 0.05, **P < 0.01, ***P < 0.001.

To determine whether the transcriptional regulator Paf1C is involved in the regulation of *N. crassa* circadian rhythm, we generated mutant strains of Paf1C subunits via homologous recombination (Fig 1A). Race tube assays showed that both *paf-1*^*KO*^ and *ctr-9*^*KO*^ strains have a slow growth rate and produce sparse conidia. More importantly, the *paf-1* or *ctr-9* deletion strains exhibited a conidiation rhythm approximately 3.5 hours shorter than the WT strain in constant darkness (DD) (Fig 1B). The amplitude of the conidiation rhythm of *paf-1*^*KO*^ and *ctr-9*^*KO*^ strains was significantly lower compared to that in WT strain, indicating that PAF-1 and CTR-9 regulate both the period and amplitude of the circadian rhythm. However, the *rtf-1*^*KO*^ strain also has a slow growth rate and exhibited ~1.5 hours shorter conidiation rhythm than the WT strain in DD, with a similar amplitude to that of the WT strain (Fig 1B). Under light/dark (LD) cycles, the phase of the conidiation rhythm in the three mutant strains was ~ 2–3 hours earlier than that of the WT strain (S1A Fig). Interestingly, the loss of CDC-73 or LEO-1 subunit had little effect on growth rate and the conidiation rhythm (Fig 1B). These results indicate that each subunit of Paf1C contributes differently to its function in the *N. crassa* circadian rhythm.

To further confirm the functions of these subunits in the circadian clock, we introduced three constructs expressing Myc-tagged PAF-1, CTR-9 or RTF-1 driven by quinic acid (QA)-inducible *qa-2* promoter into each corresponding mutant strain. In QA-containing race tubes, the conidiation rhythms of each mutant were comparable to those of the WT strains when expressing the corresponding Myc-tagged proteins (S1B Fig), indicating that Myc-tagged PAF-1, CTR-9, or RTF-1 can complement the function of the endogenous PAF-1, CTR-9, or RTF-1 protein in the *paf-1*^*KO*^, *ctr-9*^*KO*^, or *rtf-1*^*KO*^ mutant. Together, these results suggest that the transcription elongation factor Paf1C is an important regulator of the *N. crassa* circadian clock.

### The Paf1C subunits play distinct roles in controlling the normal expression of the *frq* gene

To confirm the functions of Paf1C subunits in regulating *frq* expression, we first introduced a *frq* promoter driven luciferase reporter construct (*frq-luc*) into the WT or *paf-1*^*KO*^ strain at the *his-3* locus, respectively. The bioluminescence rhythm of *paf-1*^*KO*^; *frq-luc* strain displayed lower amplitude and shorter period compared to the *wt*; *frq-luc* strain (S1C and S1D Fig), indicating that the PAF-1 is required for rhythmic expression of *frq* at the RNA level. We then compared the circadian rhythms of FRQ expression in WT and Paf1C mutant strains in DD. FRQ protein levels, along with its phosphorylation status, oscillated well in the WT strain in DD (Fig 1C). However, in the *paf-1*^*KO*^ or *ctr-9*^*KO*^ mutant, overall FRQ levels were significantly lower and FRQ oscillated with lower amplitude than those in the WT strain (Fig 1C). Similarly, *frq* mRNA levels were lower in the *paf-1*^*KO*^ or *ctr-9*^*KO*^ strain compared to the WT strain in DD (Fig 1D), suggesting that PAF-1 and CTR-9 subunits promote *frq* transcription. In the *rtf-1*^*KO*^ mutant, overall FRQ protein and *frq* mRNA levels were comparable to those in the WT strain, oscillating with a similar amplitude (Fig 1C and 1D). However, in constant light (LL, hour 0 time point), the levels of FRQ protein and *frq* mRNA in the *rtf-1*^*KO*^ strain were higher than those in the WT strain (Fig 1C and 1D), suggesting that the RTF-1 subunit suppresses *frq* transcription under LL condition.

We then assessed FRQ stability after the addition of the protein synthesis inhibitor cycloheximide (CHX) and found that FRQ degradation rate was similar in WT and these mutant strains (S1E Fig). These results further emphasize the importance of Paf1C in regulating the circadian clock in *N. crassa*, primarily by facilitating *frq* transcription rather than influencing FRQ degradation.

### The Paf1C subunits directly bind to the *frq* locus for its regulation

**Fig 2:**
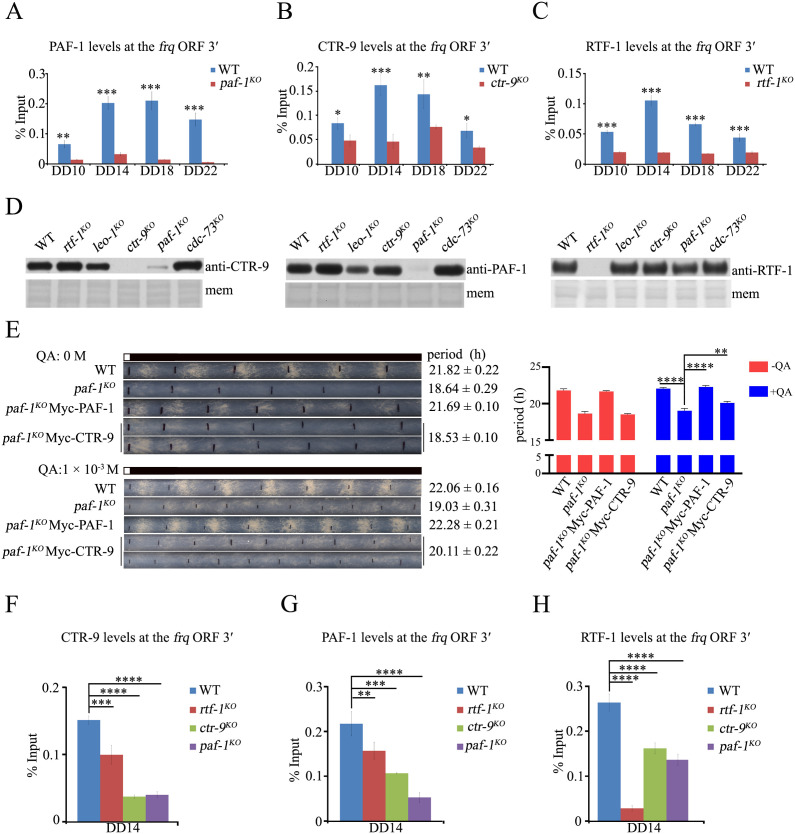
The Paf1C rhythmically binds to the *frq* locus. (A-C) ChIP analysis showing the recruitment of PAF-1 (A), CTR-9 (B) and RTF-1 (C) at the *frq* ORF3′ region at the indicated time points*.* Error bars are means ± SD. (n = 3). Significance difference was assessed by Student’s *t*-test. *P < 0.05, **P < 0.01, ***P < 0.001. (D) Western blot analysis of the levels of CTR-9 (Left), PAF-1 (Middle), RTF-1 (Right) in WT, *paf-1*^*KO*^, *ctr-9*^*KO*^, *rtf-1*^*KO*^, *leo-1*^*KO*^, *cdc-73*^*KO*^ strains. The PVDF membrane (mem) stained with Coomassie blue was used as a loading control. (E) Race tube assay (Left) and statistical analyses (Right) showing the circadian conidiation rhythms and period length of WT, *paf-1*^*KO*^, *paf-1*^*KO*^;qa-Myc-PAF-1, *paf-1*^*KO*^;qa-Myc-CTR-9 strains. The concentration of the quinic acid (QA) in race tube assay is 0 M (Upper) and 1 × 10^-3^ M (Lower). Error bars are means ± SD. (n = 3). Significance difference was assessed by Ordinary one-way ANOVA Multiple comparisons. **P < 0.01, ****P < 0.0001. (F-H) ChIP analysis showing that the enrichments of CTR-9 (F), PAF-1 (G), RTF-1 (H) at the *frq* ORF 3′ region. Error bars are means ± SD. (n = 3). Significance difference was assessed by Ordinary one-way ANOVA Multiple comparisons. **P < 0.01, ***P < 0.001, ****P < 0.0001.

The notable differences in period length and *frq* expression between *rtf-1*^*KO*^ and *paf-1*^*KO*^ or *ctr-9*^*KO*^ strains strongly suggest that RTF-1’s role in clock regulation differs from that of PAF-1 and CTR-9. To further investigate whether Paf1C subunits regulate *frq* transcription by directly binding to the *frq* locus, we generated polyclonal antibodies against endogenous PAF-1 (72 kDa), CTR-9 (155 kDa), and RTF-1 (90 kDa) proteins (S2A Fig). ChIP assays using these antibodies showed that the enrichment of PAF-1, CTR-9 or RTF-1 protein at the ORF 3′ of the *frq* gene was specific and rhythmic in the WT strain (Figs 2A–2C and S2B–S2D), indicating that Paf1C is directly recruited to the *frq* locus to regulate the rhythmic expression of the *frq* gene.

To check whether the stability of Paf1C subunits was affected by the deletion of other subunits, we measured the protein levels of PAF-1, CTR-9, and RTF-1 in WT and each mutant strain. Western blot analyses revealed that the deletion of *paf-1* resulted in decreased levels of CTR-9 protein, while the deletion of *leo-1* caused a slight reduction in both CTR-9 and PAF-1 levels (Fig 2D). However, the protein levels of RTF-1 in each deletion mutant were comparable to those in the WT strain (Fig 2D). These results strongly suggest that the similar period defect and low *frq* expression in both *paf-1*^*KO*^ and *ctr-9*^*KO*^ strains might be due to the reduced levels of CTR-9 proteins. To test this possibility, we generated a *paf-1*^*KO*^;qa-Myc-CTR-9 transformant. As shown in race tubes, the QA-induced expression of Myc-CTR-9 increased the period length by 1 hour from the *paf-1*^*KO*^ strain (19.03 ± 0.31 h) to the *paf-1*^*KO*^;qa-Myc-CTR-9 transformant (20.11 ± 0.22 h) (Fig 2E), indicating that the lack of CTR-9 protein in both *paf-1*^*KO*^ and *ctr-9*^*KO*^ strains results in a similarly shorter period and reduced *frq* expression. We then examined the enrichment of CTR-9, PAF-9, and RTF-1 at the *frq* locus at DD14, a time point when *frq* transcription reaches its peak. Consistent with the low levels of CTR-9 in *paf-1*^*KO*^ strain, the enrichment of CTR-9 at *frq* ORF 3′ at DD14 in *paf-1*^*KO*^ strains was extremely low, even comparable to that in *ctr-9*^*KO*^ strain (the negative control), when compared to the levels in WT and *rtf-1*^*KO*^ strains (Fig 2F). Meanwhile, the enrichment of PAF-1 in *ctr-9*^*KO*^ strain was significantly decreased compared to that in WT and *rtf-1*^*KO*^ strains (Fig 2G), suggesting that PAF-1 and CTR-9 enhance each other’s binding ability at the *frq* locus. Similarly, the enrichment of RTF-1 at *frq* ORF 3′ at DD14 in both *paf-1*^*KO*^ and *ctr-9*^*KO*^ strains was also reduced compared to that in the WT strain (Fig 2H). These results indicate that the binding of PAF-1 and CTR-9 at the *frq* gene is interdependent, while their binding is only slightly affected by the loss of RTF-1.

Previous work showed that *in vitro* purified *M. thermophila* Rtf1 does not join the other four-subunit complex to form Paf1C [43]. To investigate the composition of *N. crassa* Paf1C, we generated *wt*;qa-Myc- PAF-1, LEO-1, CDC-73 or RTF-1 strains, respectively. The immunoprecipitation assay showed that PAF-1, CTR-9, LEO-1 and CDC-73 interacted with each other, while no interaction between RTF-1 and any of these four subunits was detected in *N. crassa* cells in our experimental conditions (S3A–S3D Fig), strongly suggesting that RTF-1 may interact with the Paf1 core complex with lower affinity in *N. crassa* cells. Taken together, these results suggest that RTF-1 and the PAF-1-CTR-9 subunits may regulate *N. crassa* clock through different molecular mechanisms.

### WCC-dependent *frq* transcription regulates the rhythmic association of PAF-1 and RTF-1 at the *frq* ORF

**Fig 3:**
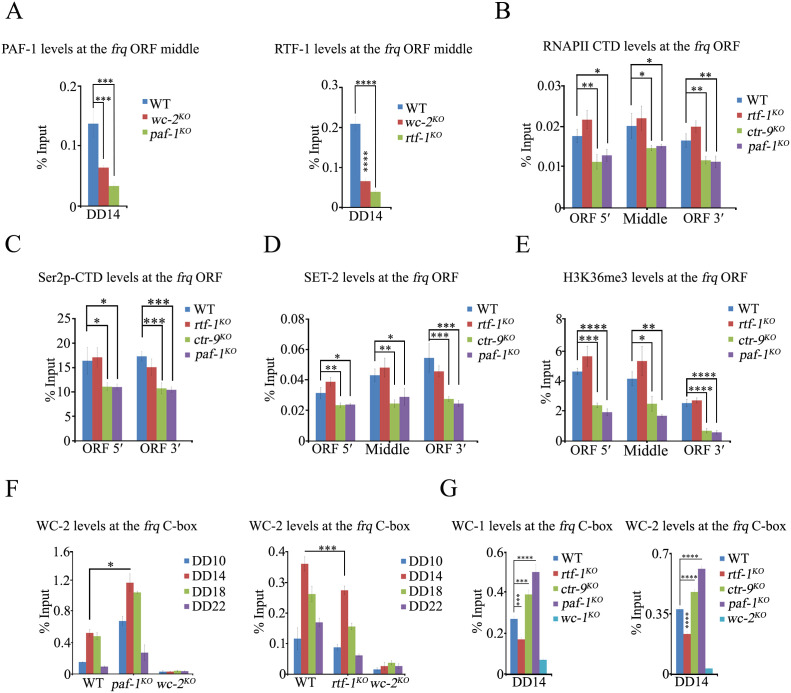
The recruitment of Paf1C to the *frq* ORF is regulated by the WCC activity. (A) The enrichment of PAF-1 (Left) or RTF-1 (Right) at the *frq* ORF middle region significantly decreased in *wc-2*^*KO*^ strain at DD14. Error bars are means ± SD. (n = 3). (B-E) ChIP analysis showing the occupancy of RNAPII CTD (B), RNAPII Ser2p (C), SET-2 (D) and H3K36me3 (E) at the *frq* ORF region in WT, *paf-1*^*KO*^, *ctr-9*^*KO*^, *rtf-1*^*KO*^ strains at DD14. Error bars are means ± SD. (n = 3). (F) ChIP analysis showing the rhythmical recruitment of WC-2 at the *frq* C-box in WT, *paf-1*^*KO*^, *rtf-1*^*KO*^ strains at the indicated time points. Error bars are means ± SD. (n = 3). Significance difference was assessed by Student’s *t*-test. *P < 0.05, ***P < 0.001. (G) ChIP analysis showing the recruitment of WCC at the *frq* C-box in WT*, paf-1*^*KO*^*, ctr-9*^*KO*^, *rtf-1*^*KO*^ strains at DD14*.* Error bars are means ± SD. (n = 3). Significance difference in Figure (A), (C-E), (G) was assessed by Ordinary one-way ANOVA Multiple comparisons. Significance difference in Figure (B) was assessed by Lognormal ordinary one-way ANOVA Multiple comparisons. *P < 0.05, **P < 0.01, ***P < 0.001, ****P < 0.0001.

Paf1C is a general transcription elongation factor of RNA polymerase II, and its binding to the *frq* ORF should depend on WCC-dependent *frq* transcription activation. We then examined the binding of PAF-1 and RTF-1 to the middle region of *frq* ORF at DD14 in WT and *wc-2*^*KO*^ strains. ChIP results revealed that the enrichment of PAF-1 and RTF-1 at the *frq* ORF was consistently low in the *wc-2*^*KO*^ strains compared to that in the WT strain (Fig 3A), confirming that the recruitment of both PAF-1 and RTF-1 to *frq* locus depends on the binding of WCC at the *frq* ORF.

To test whether the different expression levels of *frq* in *paf-1*^*KO*^, *ctr-9*^*KO*^, and *rtf-1*^*KO*^ strains are due to defects in transcription elongation, we performed ChIP assays using an RPB-1 CTD, the C-terminal domain of the largest subunit of RNA polymerase II, specific antibody to examine the recruitment of RNAPII at the *frq* gene body at DD14 in both WT and mutant strains. The ChIP data showed that the enrichment of RPB-1 at the ORF 5′, ORF Middle, ORF 3′ of the *frq* gene in *paf-1*^*KO*^ and *ctr-9*^*KO*^ strains was reduced compared to the levels in WT and *rtf-1*^*KO*^ strains (Fig 3B). To determine whether the transcription elongation of RNAPII was affected in *paf-1*^*KO*^ and *ctr-9*^*KO*^ strains, we performed ChIP assays with CTD-Ser2p, SET-2, and histone H3K36me3 specific antibodies to measure the state of RNAPII elongation on the *frq* gene body at DD14, respectively. The ChIP results showed that the Ser2p levels of RPB-1 CTD at ORF 5′ and ORF 3′ of the *frq* gene were reduced in the *paf-1*^*KO*^ and *ctr-9*^*KO*^ strains compared to the WT and *rtf-1*^*KO*^ strains (Fig 3C), indicating that the lack of PAF-1 and CTR-9, but not RTF-1 subunit, affects the transcriptional elongation of RNAPII at the *frq* gene. Furthermore, the levels of SET-2 recruitment and SET-2-mediated H3K36me3 at the ORF 5′, ORF Middle, and ORF 3′ of the *frq* gene were consistently low in *paf-1*^*KO*^ and *ctr-*9^*KO*^ strains compared to WT and *rtf-1*^*KO*^ strains at DD14 (Fig 3D and 3E). Taken together, these results indicate that PAF-1 and CTR-9 subunits, but not RTF-1, positively regulate transcription elongation of the *frq* gene during its active transcription phase.

The lower levels of FRQ protein and *frq* mRNA in *paf-1*^*KO*^ and *ctr-9*^*KO*^ strains, but not in *rtf-1*^*KO*^ strain (Fig 1C and 1D), strongly suggest that the lack of PAF-1 or CTR-9 leads to a defect in the negative feedback of FRQ to WCC activity. To test this possibility, we examined the recruitment of WC-2 at the C-box from DD10 to DD22 in WT, *paf-1*^*KO*^ and *rtf-1*^*KO*^ strains. As shown in Fig 3F, compared to the binding rhythms of WC-2 in the WT strain, robust rhythmic binding of WC-2 to the C-box was markedly increased in the *paf-1*^*KO*^ strain but slightly decreased in the *rtf-1*^*KO*^ strain, suggesting that the lack of PAF-1, but not RTF-1, affects the negative feedback loop. To further confirm this hypothesis, we performed ChIP assays using WC-1 and WC-2 antibodies, and found that the binding of WC-1 and WC-2 to the C-box at DD14 was increased in *paf-1*^*KO*^ and *ctr-9*^*KO*^ strains but decreased in the *rtf-1*^*KO*^ strain compared to the WT strain (Fig 3G), indicating that the levels of FRQ proteins in these mutants affect the binding of WCC through negative feedback.

### RAD-6 and BRE-1 function at the downstream of RTF-1 in controlling the circadian period of *N. crassa*

**Fig 4:**
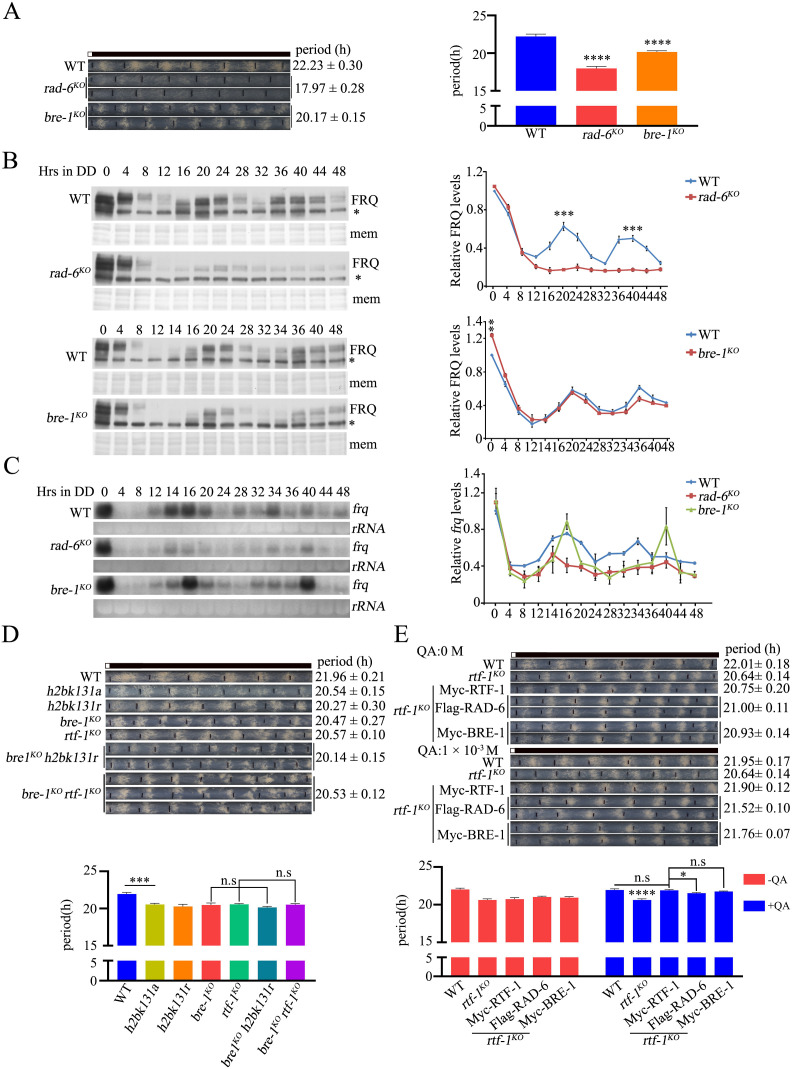
RAD-6 and Bre-1 function at the downstream of RTF-1 to regulate the normal period of the *Neurospora* circadian rhythm. (A) Race tube assay (Left) and statistical analyses (Right) showing the circadian conidiation rhythms and period length of WT, *rad-6*^*KO*^, *bre-1*^*KO*^ under constant dark. Error bars are means ± SD. (n = 3). Significance difference was assessed by Ordinary one-way ANOVA Multiple comparisons. ****P < 0.0001. (B) Western blot analysis (Left) and quantification (Right) of the levels of FRQ protein in the WT, *rad-6*^*KO*^, *bre-1*^*KO*^ strains. Asterisks indicate nonspecific bands. The PVDF membrane (mem) stained with Coomassie blue was used as a loading control. Error bars are means ± SD. (n = 3). Significance difference was assessed by Student’s *t*-*t*est. *P < 0.05, **P < 0.01, ***P < 0.001. (C) Northern blot analysis (Left) and quantification (Right) of the levels of *frq* mRNA in the WT, *rad-6*^*KO*^, *bre-1*^*KO*^ strains. The rRNA bands shown below the northern blot were used as a loading control for each sample. Error bars are means ± SD. (n = 3). (D) Race tube assays (Upper) and statistical analyses (Lower) showing the conidiation period of WT, *h2bk131a*, *h2bk131r*, *bre-1*^*KO*^, *bre-1*^*KO*^ *h2bk131r*, *rtf-1*^*KO*^, *rtf-1*^*KO*^ *bre-1*^*KO*^ strains. Error bars are means ± SD. (n = 3). Significance difference was assessed by Student’s *t*-test. n.s P ≥ 0.05, ***P < 0.001. (E) Race tube assays (Upper) and statistical analyses (Lower) showing the conidiation period of WT, *rtf-1*^*KO*^, *rtf-1*^*KO*^;qa-Myc-RTF-1, *rtf-1*^*KO*^;qa-Flag-RAD-6, *rtf-1*^*KO*^;qa-Myc-BRE-1 strains*.* The concentration of the quinic acid (QA) in race assay is 0 M (Upper) and 1 × 10^-3^M (Lower). Error bars are means ± SD. (n = 3). Significance difference was assessed by Ordinary one-way ANOVA Multiple comparisons. n.s P ≥ 0.05, *P < 0.05, ****P < 0.0001.

In yeast, the deletion of the *rtf1* gene results in the loss of global H2BK123ub mediated by Rad6-Bre1 ubiquitin conjugase-ligase complex, which affects the expression of numerous genes [56,58]. Previous studies indicate that the depletion of Rnf20, the global E3 ligase for H2BK120ub in mammals, shortens circadian period length [37], while disruption of *hub1*, which encodes the E3 ubiquitin ligase HUB1, exhibited shorter circadian period and advanced phases compared to the wild type *Arabidopsis* [38]. To test whether the shorter conidiation period of *paf-1*^*KO*^, *ctr-9*^*KO*^, and *rtf-1*^*KO*^ strains are due to the loss of H2Bub, we created deletion strains of *rad-6* (NCU09731) and *bre-1* (NCU07544) in *N. crassa*, respectively. As shown in Fig 4A, the *rad-6* deletion strain exhibited a shorter conidiation period (17.97 ± 0.28 h) in a race tube assay compared to the WT strain (22.23 ± 0.3 h). Interestingly, similarly to *paf-1*^*KO*^ and *ctr-9*^*KO*^ strains, the *rad-6*^*KO*^ strain showed a slow growth rate and produced fewer conidia (Fig 4A). The *bre-1*^*KO*^ strain also has a slow growth rate and exhibited a similar shorter period of conidiation rhythm (20.17 ± 0.15 h) compared to the *rtf-1*^*KO*^ strain (Fig 4A). Under LD cycles, the phase of the conidiation rhythm of *rad-6*^*KO*^ or *bre-1*^*KO*^ strain was ~ 3 h earlier than that of the WT strain (S4A Fig). Furthermore, the period length of *rad-6*^*KO*^;qa-Flag-RAD-6 or *bre-1*^*KO*^;qa-Myc-BRE-1 strain was restored to that of WT strains by expressing the Flag-RAD-6 or Myc-BRE-1 protein in each corresponding mutant in race tube with QA (S4B Fig), suggesting that the H2Bub is a highly conserved mechanism for regulating circadian clock in eukaryotes.

To determine how RAD-6 and BRE-1 influence the circadian clock, we examined the FRQ protein and *frq* mRNA expression profiles in DD. Like the *paf-1*^*KO*^ and *ctr-9*^*KO*^ strains, FRQ protein and *frq* mRNA levels were lower in the *rad-6*^*KO*^ strain than those in the WT strain in DD (Fig 4B and 4C), suggesting that RAD-6 promotes *frq* transcription. Interestingly, the levels of FRQ protein and *frq* mRNA in the *bre-1*^*KO*^ strain were similar to those in the WT strain in DD, but higher than those in the WT strain in LL (Fig 4B and 4C). Although the *rad-6*^*KO*^ and *bre-1*^*KO*^ strains exhibited different developmental and clock phenotypes, these results indicate that RAD-6 and BRE-1 are required for the proper functioning of the *N. crassa* clock.

Amino acid alignment suggested that the lysine at position 131 of *N. crassa* H2B is the ubiquitylated site, which is evolutionarily conserved in budding yeast and humans (S4C Fig). To investigate whether the RAD-6-BRE-1-mediated H2BK131ub plays a role in maintaining period length, we generated *h2bk131r* and *h2bk131a* single mutants using the knock-in method, along with *bre-1*^*KO*^ *h2bk131r* and *bre-1*^*KO*^ *rtf-1*^*KO*^ double mutants. As shown in Fig 4D, both *h2bk131r* and *h2bk131a* mutants have a slow growth rate and exhibited a similar shorter period compared to those of *bre-1*^*KO*^, *rtf-1*^*KO*^, *bre-1*^*KO*^ *h2bk131r* or *bre-1*^*KO*^ *rtf-1*^*KO*^ strains, confirming that RTF-1 and BRE-1 regulate the conidiation period through the H2BK131ub pathway. To further test the genetic interaction, we introduced a qa-Myc-BRE-1 or a qa-Flag-RAD-6 into *rtf-1*^*KO*^ strains. Indeed, QA-induced ectopic expression of Myc-BRE-1 or Flag-RAD-6 restored the shorter period of *rtf-1*^*KO*^ mutants to nearly wild-type period length in *rtf-1*^*KO*^;qa-Myc-BRE-1 or *rtf-1*^*KO*^;qa-Flag-RAD-6 transformants (Fig 4E). However, ectopic expression of Flag-RAD-6 or Myc-BRE-1 in *paf-1*^*KO*^ strains failed to restore its shorter period (S5A Fig). Additionally, we performed an immunoprecipitation assay to test the interaction between RTF-1 and BRE-1 in *wt*;qa-Myc-RTF-1 or *wt*;qa-Myc-BRE-1 transformants. Co-IP results showed that RTF-1 interacted with BRE-1 in *N. crassa* cells (S5B Fig). Taken together, these results demonstrate that the RAD-6-BRE-1 functions at the downstream of RTF-1 to regulate *N. crassa* circadian rhythm via the H2BK131ub pathway.

### Both HMD and Plus3 domains of RTF-1 play key roles in regulating the circadian rhythm

**Fig 5:**
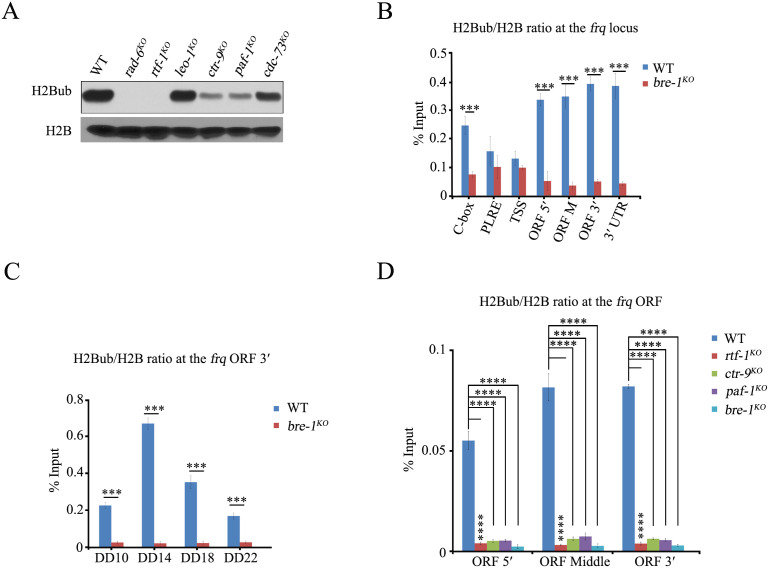
H2B is rhythmically ubiquitinated at the *frq* locus. (A) Western blots analysis of the levels of global H2Bub and H2B in WT, *rad-6*^*KO*^, *rtf-1*^*KO*^, *leo-1*^*KO*^, *ctr-9*^*KO*^, *paf-1*^*KO*^, *cdc-73*^*KO*^ strains. (B-C) ChIP analysis showing the enrichment of H2Bub/H2B ratio at different regions of the *frq* locus at DD14 (B) and at the *frq* ORF3′ at the indicated time points (C) in WT, *bre-1*^*KO*^ strains. Error bars are means ± SD. (n = 3). Significance difference was assessed by Student’s *t*-test. *P < 0.05, **P < 0.01, ***P < 0.001. (D) ChIP analysis showing the levels of H2Bub/H2B ratio at the *frq* ORF regions in WT, *rtf-1*^*KO*^, *paf-1*^*KO*^, *ctr-9*^*KO*^, *bre-1*^*KO*^ strains. Error bars are means ± SD. (n = 3). Significance difference was assessed by Ordinary one-way ANOVA Multiple comparisons. ****P < 0.0001.

To investigate the roles of Paf1C subunits in histone H2B ubiquitylation in *N. crassa* cells, we assessed the global H2B monoubiquitylation of WT, *rad-6*^*KO*^, *paf-1*^*KO*^, *ctr-9*^*KO*^, *rtf-1*^*KO*^, *cdc-73*^*KO*^, and *leo-1*^*KO*^ strains. Western blot analysis showed that the loss of *rtf-1* or *rad-6* gene abolished H2BK131ub compared to WT strains (Fig 5A). However, the levels of H2BK131ub in *ctr-9*^*KO*^ and *paf-1*^*KO*^ strains were dramatically reduced compared to those in WT, *cdc-73*^*KO*^, and *leo-1*^*KO*^ strains (Fig 5A). We next measured the levels of H2BK131ub at the *frq* locus in WT and *bre-1*^*KO*^ strains. ChIP assays revealed that H2BK131ub occurred at the C-box, ORF 5′, ORF Middle, and ORF 3′ of the *frq* gene in WT strains but not in *bre-1*^*KO*^ strains at DD14 (Fig 5B). During *frq* transcription, histone H2B at the *frq* ORF was rhythmically ubiquitylated in the WT strain, but this modification was completely abolished in *bre-1*^*KO*^ strains (Fig 5C). Similarly to *bre-1*^*KO*^ strains, the levels of H2BK131ub at the *frq* ORF in *rtf-1*^*KO*^, *paf-1*^*KO*^, and *ctr-9*^*KO*^ strains were dramatically reduced compared to those in the WT strain (Fig 5D), indicating that Paf1C facilitates H2BK131ub at the *frq* locus during the transcription elongation of the *frq* gene.

**Fig 6:**
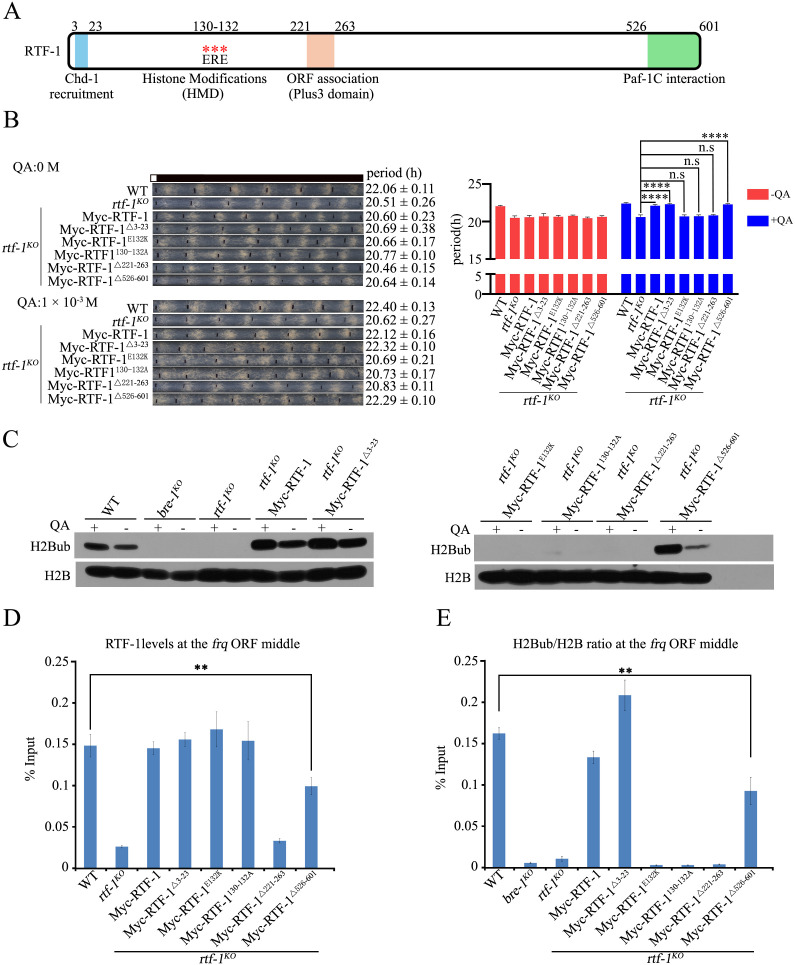
The HMD and Plus3 domains within RTF-1 regulate the circadian conidiation period and H2B ubiquitination at the *frq* locus. (A) Diagram showing the conserved domain of RTF-1. The Light blue rectangle (amino acid 3-23) indicates CHD-1 recruitment domain; the red asterisks (amino acid 130-132) represent ERE residues which are required for RTF-1 to promote histone modifications; the aurantia rectangle (amino acid 221-263) indicates the Plus3 domain; the green rectangle (amino acid 526-601) represents Paf1C interaction domain. (B) Race tube assays (Left) and statistical analyses (Right) showing the conidiation period of WT, *rtf-1*^*KO*^, *rtf-1*^*KO*^;qa-Myc-RTF-1, *rtf-1*^*KO*^;qa-Myc-RTF-1^△3-23^, *rtf-1*^*KO*^;qa-Myc-RTF-1^E132K^, *rtf-1*^*KO*^;qa-Myc-RTF-1^130-132A^, *rtf-1*^*KO*^;qa-Myc-RTF-1^△221-263^, *rtf-1*^*KO*^;qa-Myc-RTF-1^△526-601^ strains*.* The concentration of the quinic acid (QA) in race tube assay is 0 M (Upper) and 1 × 10^-3^ M (Lower). Error bars are means ± SD. (n = 3). Significance difference was assessed by Ordinary one-way ANOVA Multiple comparisons. n.s P ≥ 0.05, ****P < 0.0001. (C) Western blots showing the global levels of H2Bub in WT, *bre-1*^*KO*^, *rtf-1*^*KO*^, *rtf-1*^*KO*^; qa-Myc-RTF-1, *rtf-1*^*KO*^;qa-Myc-RTF-1^△3-23^, *rtf-1*^*KO*^;qa-Myc-RTF-1^E132K^, *rtf-1*^*KO*^; qa-Myc-RTF-1^130-132A^, *rtf-1*^*KO*^;qa-Myc-RTF-1^△221-263^, *rtf-1*^*KO*^;qa-Myc-RTF-1^△526-601^ strains. (D-E) ChIP assays showing the enrichment of RTF-1 (E) or H2Bub/H2B ratio (F) at the *frq* ORF middle region in the WT, *rtf-1*^*KO*^, *rtf-1*^*KO*^;qa-Myc-RTF-1, *rtf-1*^*KO*^;qa-Myc-RTF-1^△3-23^,*rtf-1*^*KO*^;qa-Myc-RTF-1^E132K^, *rtf-1*^*KO*^;qa-Myc-RTF-1^130-132A^, *rtf-1*^*KO*^;qa-Myc-RTF-1^△221-263^, *rtf-1*^*KO*^;qa-Myc-RTF-1^△526-601^ strains*.* The working concentration of QA in ChIP assays is 1 × 10^-3^ M. Error bars are means ± SD. (n = 3). Significance difference was assessed by Student’s *t*-*t*est. **P < 0.01.

Previous studies have shown that specific separation-of-function alleles have been reported for yeast Rtf1, highlighting regions important for Chd1 recruitment, histone modifications (HMD), ORF association (Plus3 domain), and Paf1C interaction [63,64]. Alignment with other Rtf1 homologs showed that *N. crassa* RTF-1 is more closely related to the Rtf1 of budding yeast than to those of higher eukaryotes (*Homo sapiens* and *Drosophila melanogaster*) (S6A Fig). To determine which domains of RTF-1 play key roles in regulating the *N. crassa* circadian rhythm, we constructed several p*qa-2-*Myc-RTF-1 plasmids with mutations or deletions in the RTF-1 coding region necessary for Chd1 recruitment (RTF1^Δ3-23^), H2BK131ub (RTF-1^E132K^, RTF-1^130-132A^), ORF association (Plus3 domain) (RTF-1^Δ221-263^), or Paf1C interaction (RTF-1^Δ526-601^) (Fig 6A) and expressed each plasmid in a *rtf-1*^*KO*^ strain, respectively. Immunoblot analysis demonstrated that each Myc-RTF-1 mutant protein was expressed in response to QA (S6B Fig). As shown in Fig 6B, in QA-containing race tubes, the shorter period of the *rtf-1*^*KO*^ strain was rescued to that of the WT strain by expressing Myc-RTF-1, RTF-1^Δ3-23^, or RTF-1^Δ526-601^ proteins, whereas the expression of Myc-RTF-1^E132K^, RTF-1^130-132A^ or RTF-1^Δ221-263^ proteins cannot restore the shorter period of the *rtf-1*^*KO*^ strain. Consistent with the clock phenotypes of these transformants, ectopic expression of Myc-RTF-1^E132K^, RTF-1^130-132A^, or RTF-1^Δ221-263^ proteins cannot restore the global H2BK131ub in the *rtf-1*^*KO*^ strain (Fig 6C), whereas expression of Myc-RTF1, RTF1^Δ3-23^, or RTF1^Δ526-601^ proteins fully restored the H2BK131ub in the *rtf-1*^*KO*^ strain (Fig 6C). These findings suggest that both the HMD and Plus3 domains of RTF-1 are required for circadian clock function by regulating histone H2BK131ub.

We then measured the enrichment of Myc-RTF-1 proteins and the H2BK131ub levels at the *frq* locus. As shown in Fig 6D, deletion of Plus3 domain (RTF-1^Δ221-263^) but not other mutations of RTF-1 completely abolished RTF-1 binding to the *frq* ORF region. Furthermore, ChIP assays revealed that the binding of Myc-RTF-1 or RTF-1^Δ3-23^ but not any other of RTF-1 HMD mutants in *rtf-1*^*KO*^ strains fully restored the H2BK131ub levels at the *frq* locus to those of the WT strain (Fig 6E), although the decreased binding of Myc-RTF1^Δ526-601^ partially restored the defect of H2BK131ub in the *rtf-1*^*KO*^ strain compared to those in the WT strain (Fig 6E). Taken together, these results indicate that both HMD and Plus3 domain of RTF-1, but not its Chd1 recruitment and Paf1C interaction domains, are important for H2B ubiquitylation, which determines proper clock function.

### The HMD domain of RTF-1 is both necessary and sufficient for the proper function of RTF-1 in regulating the circadian rhythm

**Fig 7:**
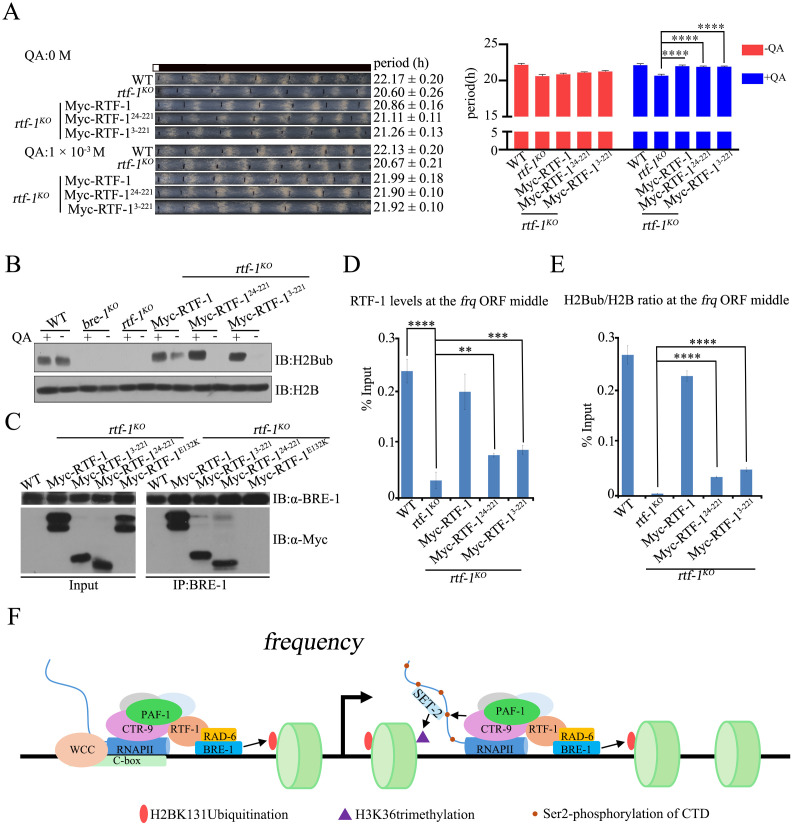
The HMD domain is necessary and sufficient for proper function of RTF-1 in regulation of circadian period. (A) Race tube assays (Left) and statistical analyses (Right) showing the circadian conidiation period of WT, *rtf-1*^*KO*^, *rtf-1*^*KO*^;qa-Myc-RTF-1, *rtf-1*^*KO*^;qa-Myc-RTF-1^24-221^, *rtf-1*^*KO*^;qa-Myc-RTF-1^3-221^ strains*.* The concentration of the quinic acid (QA) in race assay is 0 M (Upper) and 1 × 10^-3^M (Lower). Error bars are means ± SD. (n = 3). Significance difference was assessed by Ordinary one-way ANOVA Multiple comparisons. ****P < 0.0001. (B) Western blots analysis of the global levels of H2Bub in WT, *bre-1*^*KO*^, *rtf-1*^*KO*^, *rtf-1*^*KO*^;qa-Myc-RTF-1, *rtf-1*^*KO*^;qa-Myc-RTF-1^24-221^, *rtf-1*^*KO*^;qa-Myc-RTF-1^3-221^strains. (C) Co-IP assay showing BRE-1 interacts with Myc-tagged RTF-1, RTF-1^3-221^, RTF-1^24-221^ but not with RTF-1^E132K^. (D-E) ChIP assays showing the enrichment of RTF-1^WT^, RTF-1^24-221^, RTF-1^3-221^ (D) or H2Bub/H2B ratio (E) at the *frq* ORF middle in WT, *rtf-1*^*KO*^, *rtf-1*^*KO*^;qa-Myc-RTF-1, *rtf-1*^*KO*^;qa-Myc-RTF-1^24-221^, *rtf-1*^*KO*^;qa-Myc-RTF-1^3-221^strains*.* The concentration of QA in ChIP assays is 1 × 10^-3^M. Error bars are means ± SD. (n = 3). Significance difference was assessed by Lognormal ordinary one-way ANOVA Multiple comparisons. **P < 0.01, ***P < 0.001, ****P < 0.0001. (F) The working model illustrating the role of Paf1C in regulating the expression of the circadian clock gene *frq* in *Neurospora*. Paf1C maintains a normal circadian clock by promoting RAD-6/BRE-1-mediated histone H2B ubiquitination and facilitating Ser2 phosphorylated RNAPII CTD, as well as SET-2-mediated H3K36 trimethylation at the *frq* locus in *Neurospora*.

The dispensable function of Paf1C interaction domain in RTF-1 for clock regulation strongly suggests that maintaining H2BK131ub at the *frq* locus plays an important role in the proper functioning of circadian rhythms in *N. crassa*. In yeast, the expression of Rtf1 HMD in *rtf1Δ* cells is sufficient to promote Paf1C-dependent histone modifications [58,59]. Therefore, we constructed qa-Myc-RTF-1^(24–221aa)^ and qa-Myc-RTF-1^(3–221aa)^ plasmids that include the HMD domain (24–221aa) and generated the *rtf-1*^*KO*^;qa-Myc-RTF-1^(24–221aa)^ and *rtf-1*^*KO*^;qa-Myc-RTF-1^(3–221aa)^ transformant strains. As shown in Fig 7A, the expression of Myc-RTF-1^(24–221aa)^ or Myc-RTF-1^(3–221aa)^ can restore the short period phenotypes of the *rtf-1*^*KO*^ strain to nearly the WT period length in QA-containing race tubes, indicating that the RTF-1 HMD is necessary and sufficient for RTF-1 in regulating the conidiation rhythm. Western blot analysis revealed that the expression of Myc-RTF-1, RTF-1^(24–221aa)^, or RTF-1^(3–221aa)^ proteins fully restored the low levels of H2BK131ub in the *rtf-1*^*KO*^ strain to those in the WT strain (Fig 7B). Immunoprecipitation assay showed that Myc-RTF-1, RTF-1^(24–221aa)^, or RTF-1^(3–221aa)^, but not Myc-RTF-1^E132K^ protein, interacted with BRE-1 in transformant cells (Fig 7C), confirming that the expression of RTF-1 HMD in *rtf-1*^*KO*^ cells is sufficient to promote Paf1C-dependent H2BK131ub in *N. crassa*.

To further confirm whether the expression of RTF-1 HMD in *rtf-1*^*KO*^ strains also promotes Paf1C-dependent H2BK131ub at the *frq* locus, we measured the enrichment of Myc-RTF-1 HMD proteins and H2BK131ub at the *frq* locus, respectively. ChIP assay showed that the levels of Myc-RTF-1 binding at the *frq* locus in *rtf-1*^*KO*^;qa-Myc-RTF-1 strains were similar to those in the WT strain, while the binding levels of Myc-RTF-1^(24–221aa)^ or Myc-RTF-1^(3–221aa)^ proteins significantly decreased in the transformants (Fig 7D). Consistent with these results, the decreased binding of Myc-RTF-1^(24–221aa)^ or Myc-RTF-1^(3–221aa)^ proteins at the *frq* locus partially restored the low levels of H2BK131ub at the *frq* locus in the *rtf-1*^*KO*^ strain compared to those in WT and *rtf-1*^*KO*^;qa-Myc-RTF-1 strains (Fig 7E). Taken together, both genetic and biochemical data demonstrate that the low levels of H2BK131ub mediated by RTF-1 HMD at the *frq* locus are sufficient for the proper regulation of circadian conidiation rhythms in *N. crassa*.

## Discussion

In the *N. crassa* circadian system, the running of the central oscillator is based on a transcription-translational negative feedback loop. Therefore, the precise regulation of *frq* transcription is a critical process for the operation of the *Neurospora* circadian clock [65]. Although we have a solid understanding of the transcriptional activation and repression of *frq* expression, the role of transcription elongation factors in *frq* regulation remains less understood. The evidence presented here indicated that Paf1C, a well-known transcription elongation factor complex, accurately regulates *Neurospora* circadian period by facilitating transcription elongation and H2BK131ub at the *frq* locus (Fig 7F). Firstly, the deletion of *paf-1*, *ctr-9* or *rtf-1* resulted in shorter circadian periods of *N. crassa* conidiation rhythm (Fig 1B). Secondly, Paf1C rhythmically binds to the *frq* locus, which depends on the WCC-activated *frq* transcription (Figs 2A–2C and 3A). Thirdly, the disruption of H2BK131ub in the *rtf-1*^*KO*^, *rad-6*^*KO*^, *bre-1*^*KO*^, or *h2bk131r/a* strain led to shorter circadian periods (Fig 4A and 4D) and the shortened period in the *rtf-1*^*KO*^ strain can be restored by ectopic expression of RAD-6 or BRE-1 (Fig 4E). Finally, the expression of RTF-1 HMD can rescue global H2BK131ub and the conidiation period in the *rtf-1*^*KO*^ strain (Fig 7A and 7B). Taken together, our results indicate that Paf1C maintains the normal circadian period by regulating both the transcriptional elongation and the H2BK131ub at the *frq* locus.

Although Paf1C contains five subunits, previous studies have shown that each subunit contributes unequally to Paf1C’s function [42,43]. In this study, the deletion of *paf-1* or *ctr-9* led to a ～ 18.5 hours circadian period and reduced conidiation, while the deletion of *rtf-1* resulted in a ～ 20.5 hours circadian period with normal conidiation. Moreover, the deletion of *cdc-73* or *leo-1* had no effect on period length and development (Fig 1B). These genetic results strongly suggest that the PAF-1 and CTR-9 subunits play a more significant role in regulating circadian clock than the RTF-1 subunit in Paf1C. Western blot analysis revealed that the protein levels of CTR-9 nearly disappeared in the *paf-1*^*KO*^ strain but remained in other Paf1C subunit mutants (Fig 2D), indicating that the loss of the CTR-9 and PAF-1 activities accelerates the clock compared to the *rtf-1*^*KO*^ strain and WT strain. Consistent with these results, the binding levels of CTR-9 and PAF-1 at the *frq* ORF at DD14 are interdependent, indicating that PAF-1 and CTR-9 affect each other’s binding ability at the *frq* locus (Fig 2F and 2G). Furthermore, ectopic expression of Myc-CTR-9 in *paf-1*^*KO*^;qa-Myc-CTR-9 strain nearly rescued the conidiation period of the *paf-1*^*KO*^ strain (Fig 2E). Taken together, these results confirm that the lack of CTR-9 protein in both *paf-1*^*KO*^ and *ctr-9*^*KO*^ strains, but not in the *rtf-1*^*KO*^ strain, results in a significantly shorter period and lower amplitude of *frq* expression.

In yeast, the deletion of the *rtf1* gene results in the loss of global H2BK123ub mediated by the Rad6-Bre1 ubiquitin conjugase-ligase complex, which affects the expression of many genes [56,58]. Our ChIP results revealed that H2BK131ub was rhythmic at the *frq* locus in the WT strain but was significantly reduced across the entire gene in *paf-1*^*KO*^, *ctr-9*^*KO*^, and *rtf-1*^*KO*^ strains (Fig 5B–D), indicating that H2BK131ub is involved in the functioning of the circadian clock in *N. crassa*. The shortened period and complete absence of H2BK131ub in *rtf-1*^*KO*^ indicate that RAD-6-BRE-1-mediated H2BK131ub plays a crucial role in controlling period length by modifying the chromatin of clock genes. Disruption of H2BK131ub in the *rad-6*^*KO*^ strains significantly shortened the period length and decreased the amplitude of *frq* expression, similar to the *paf-1*^*KO*^ or *ctr-9*^*KO*^ strains (Fig 4A and 4B). As with RTF-1, the disruption of H2BK131ub in the *bre-1*^*KO*^, *h2bk131r* or *h2bk131a* strains resulted in a period shortening of approximately 1.5 hours, while the amplitude of *frq* expression was not affected. Our genetic data indicated that the conidiation periods of *bre-1*^*KO*^ *rtf-1*^*KO*^ double mutants resembled those of either *bre-1*^*KO*^ or *rtf-1*^*KO*^ strains (Fig 4D), indicating that RTF-1 and BRE-1 function in the same pathway. Previous studies have shown that yeast Rtf1 HMD alone is sufficient to promote Paf1C-dependent H2BK123ub in yeast [58]. Consistently, ectopic expression of Myc-RTF-1^24-221aa^ within RTF-1, which is similar to yeast HMD (S6A Fig), rescued the circadian period and global H2BK131ub in the *rtf-1*^*KO*^ strain (Fig 7A and 7B). Yeast HMD associates with the chromatin of the *PYK1* and *PMA1* genes [58]. Despite significantly reduced binding levels of RTF-1^24-221^ at the *frq* locus, the H2BK131ub levels at this locus were partially rescued (Fig 7D and 7E). In *Arabidopsis*, histone H2B monoubiquitination is required for achieving maximal transcript levels of the circadian clock gene, *CCA1* [66]. Mutation of the E3 ubiquitin ligase HUB1, and its associated protein SPEN3 resulted in ～1 hour period shortening and an advanced phase [38,66]. Although these Paf1C mutants and *rad-6*^*KO*^, *bre-1*^*KO*^, *h2bk131r* strains showed different degrees of period shortening, all mutants displayed an advanced phase of approximately 2–3 hours compared to WT strains. These results strongly suggest that the loss of H2BK131ub contributed to only about ~1.5 hours of circadian period length and 2–3 hours of phase regulation in *N. crassa*. Furthermore, in the *A. thaliana hub1–4* and *spen3–1* mutants, the reduced gene expression correlated with a significant decrease in H2Bub at the clock gene *CCA1* body [38]. In mammalian cells, disruption of H2Bub at both *Per1* and *Per2* E-box sites by depletion of either Ddb1 or Cul4a protein that interacts with Clock-Bmal1, or by depletion of Rnf20, a global E3 ligase for H2Bub, caused a shortened circadian period length [37]. Taken together, these results indicate that the regulation of circadian clock by H2Bub is a highly conserved mechanism among different eukaryotic species.

If the loss of H2BK131ub in Paf1C mutants contributed to only about ~1.5 hours of circadian period length and 2–3 hours phase regulation, the shorter period and lower amplitude of *frq* expression in *paf-1*^*KO*^ and *ctr-9*^*KO*^ strains indicate that PAF-1 and CTR-9 subunits play more significant roles in *frq* regulation than the RTF-1 subunit. Our previous study showed that the loss of SET-2-mediated H3K36me3 results in defects in circadian clock and *frq* expression [36]. Here, our ChIP data revealed a marked reduction in the enrichment of Ser2 phosphorylated RNAPII CTD, SET-2 and H3K36me3 at the *frq* ORF region in *paf-1*^*KO*^ and *ctr-9*^*KO*^ strains, with no detectable change in their enrichments at the *frq* ORF region in *rtf-1*^*KO*^ strain (Fig 3C–3E). SET-2-mediated H3K36me3 is a hallmark of RNAPII transcription elongation, so *frq* transcription elongation is severely impaired in *paf-1*^*KO*^ and *ctr-9*^*KO*^ strain but not in the *rtf-1*^*KO*^ strain (Fig 3C–3E). Similarly, the loss of Paf1 or Ctr9 subunit led to a significant reduction of H3K36me3, whereas no detectable H3K36me3 defect was observed in the yeast *rtf1Δ* strain [53]. These biochemical data demonstrate functional distinctions among the subunits of the Paf1C. Structural analyses revealed that yeast Paf1 and Ctr9 form a heterodimer, which functions as a scaffold to hold Cdc73, Leo1 and Rtf1 together [42,67]. Structural studies from *M. thermophile* indicate that Paf1, Ctr9 and Cdc73 form a trimer, where Ctr9 wraps around the N-terminal of Paf1, and Cdc73 is embedded in a surface groove of Ctr9 [43]. Consistent with this observation, Co-IP results indicated that RTF-1 failed to interact with other Paf1C subunits in *N. crassa* (S3A–S3D Fig), suggesting that RTF-1 might do not form a complex with other subunits, or interact with the Paf1 core complex with lower affinity in *N. crassa*. Taken together, our study depicted that RTF-1 mainly regulates circadian period through the RAD-6/BRE-1/H2Bub signal axis. However, the loss of PAF-1 or CTR-9 dramatically reduced not only H2BK131ub levels, but also the enrichment of Ser2 phosphorylated RNAPII CTD and SET-2-mediated H3K36me3 at the *frq* locus (Fig 7F).

## Materials and methods

### Strains, culture conditions and race tube assays

The 87–3 (*bd*, *a*) strain was used as the wild-type strain in the present study. The *paf-1*^*KO*^ (*bd*, *a*), *ctr-9*^*KO*^ (*bd*, *a*), *rtf-1*^*KO*^ (*bd*, *a*), *leo-1*^*KO*^ (*bd*, *a*), *cdc7–3*^*KO*^ (*bd*, *a*), *bre-1*^*KO*^ (*bd*, *a*) or *rad-6*^*KO*^ (*bd*, *a*) was constructed by substituting the whole ORF with hygromycin resistance gene (*hph*) on the *Ku70*^*RIP*^ (*bd*, *a*) background strain [14]. To construct the *h2bk131r/a* knock-in mutant, a mutated histone *h2b* cassette containing a hygromycin resistance gene inserted downstream of the *h2b* 3′-UTR was transformed into the *Ku70*^*RIP*^ (*bd*, *a*) background strain and the transformants were selected by the hygromycin B [68]. The homokaryotic strain was screened by conidia purification and the point mutation was confirmed through DNA sequencing. The *wc-1*^*KO*^ and *wc-2*^*KO*^ strains constructed previously [69,70] were used in this research. The *rtf-1*^*KO*^ *bre-1*^*KO*^ or *bre-1*^*KO*^ *h2bk131r* double mutants were obtained by crossing.

Liquid culture conditions were the same as described previously [71–73]. For the expression of quinic acid (QA) induced protein, 1.0 × 10^-2^ M QA was added into the liquid medium containing 1 × Vogel’s medium, 0.1% glucose, and 0.17% arginine.

The medium of race tube assay contained 1 × Vogel’s salts, 0.1% glucose, 0.17% arginine, 50ng/mL biotin and 1.5% agar with or without QA.

### Plasmid

The full-length ORF and the 3′-UTR for PAF-1, CTR-9, RTF-1, LEO-1, CDC-73, BRE-1 or RAD-6 protein were amplified from the WT genomic DNA by PCR and subcloned into the qa-Myc-His or qa-Flag empty vector. The qa-Myc-His-RTF-1^E132K^ and qa-Myc-His-RTF-1^130-132A^ were generated through site-directed mutagenesis using qa-Myc-His-RTF-1 as the PCR template. The qa-Myc-His-RTF-1^△3-23^, qa-Myc-His-RTF-1^△222-263^ and qa-Myc-His-RTF-1^△536-601^ constructs were created with the corresponding primers through the same method. The point mutation or deletion was confirmed by DNA sequencing. All those plasmids were used for *his-3* targeting transformation in the *paf-1*^*KO*^ *his-3*, *rtf-1*^*KO*^ *his-3*, *ctr-9*^*KO*^ *his-3*, *bre-1*^*KO*^ *his-3* or *rad-6*^*KO*^ *his-3* strain.

### The luciferase reporter assay

The luciferase reporter assay was carried out as reported previously [33,74–76]. The luciferase reporter construct was respectively transformed into the 301–6 (*bd*, *his-3*) or *paf-1*^*KO*^ (*bd*, *his-3*) strain to get the WT; *frq-luc* or *paf-1*^*KO*^; *frq-luc* strain. The LumiCycle (ACTIMETRICS) and autoclaved FGS (Fructose-Glucose-Sucrose) – Vogel’s medium containing the 0.05% glucose, 0.05% fructose, 2% sorbose, 1 × Vogel’s medium, 50 μg/L biotin and 1.8% agar were used to carried out the luciferase assay. The conidia suspensions placed on the autoclaved FGS-Vogel’s medium supplied with 50 μM firefly D-luciferin were grown in constant light overnight. Then the cultures were transferred into constant darkness. Using the LumiCycle, the luminescence was recorded in real time after 24 hours in DD. Then the data were normalized with LumiCycle Analysis software by subtracting the baseline luciferase signal which increases as cells grow.

### Generation of the antiserums against the PAF-1, CTR-9, RTF-1 and BRE-1

GST-PAF-1 (PAF-1 amino acids 99–320), GST-CTR-9 (CTR-9 amino acids 1081–1232), GST-RTF-1 (RTF-1 amino acids 84–190) and GST-BRE-1 (BRE-1 amino acids 61–180) fusion proteins were expressed in BL21 cells. Then the recombinant proteins were purified and used as the antigen to generate rabbit polyclonal antiserum as described previously [71,72]. The antibodies against SET-2, WC-1 and WC-2 used in this study were described previously [29,36].

### Protein and RNA assay

Protein extraction, quantification and western blot analysis were performed as described previously [60,72]. For Western blot analysis, equal amounts of total protein (40μg) were loaded into each protein lane. After electrophoresis, proteins were transferred onto the PVDF membrane which was subjected to Western blot analysis with the corresponding antibodies. The antibodies used in this study for Western blots were as follows: mouse anti-Myc (Santa Cruz; 9E10; 1:3000), mouse anti-Flag (Sigma; F3165; 1:5000), rabbit anti-RTF-1(1:20000), rabbit anti-CTR-9 (1:5000), rabbit anti-PAF-1 (1:10000), rabbit anti-H2Bub (Cell signaling; 5546S; 1:2000) and rabbit anti-H2B (abcam; ab1790; 1:2000). RNA was extracted and analyzed by Northern blots as shown previously [14,17,74]. To perform Northern blots, the equal amount of total RNA (20μg) was loaded into agarose gels for electrophoresis. Then the RNA was transferred onto the Nylon membrane (GE Healthcare; RPN303B). The membrane was probed with ^32^P-UTP-labeled RNA special probes for *frq*. The T7 RNA polymerase was used to make RNA probes through *in vitro* transcription assay. For Western blot and Northern blot analyses, each experiment was carried out at least 3 times.

### Co-IP analyses

IP analyses were performed as previously described [61]. Briefly, the *Neurospora* proteins were extracted as described above. For each immunoprecipitation reaction, 2 mg protein and 5 μL Myc antibody were used [28]. After incubation with antibody for 4 hours at 4°C, 40 μL Sepharose beads were added into the samples which were incubated at 4°C for another 1 hour. The immunoprecipitates were washed three times for 5 min each with the extraction buffer before they were subjected to Western blot analysis.

### ChIP assay

ChIP experiments were performed as shown previously [61,74]. Briefly, the tissues grown in the shaking incubator were fixed with 1% formaldehyde for 15 mins at 25°C in constant darkness. The cross-link reaction was then stopped by glycine at a final concentration of 125mM for 5 mins. After that, the tissues were dried with a vacuum pump, grinded with liquid nitrogen and dissolved with 0.5g/6mL lysis buffer containing protease inhibitors (1mM PMSF, 1μg/ml leupeptin, 1μg/m pepstatin A). The chromatin DNA was broken up to ~500 bp fragments by sonication. Each immunoprecipitation reaction was performed with 1ml of protein samples (2mg/ml), and the input DNA was made from 10 μL of samples. The ChIP assay was performed with 3 μL of RTF-1 antibody, 3 μL of PAF- 1 antibody, 5 μL of CTR-9 antibody, 10 μL of SET-2 antibody, 5 μL of H2Bub antibody (Cell signaling; 5546S), 2 μL of H2B antibody (abcam; ab1790), 3 μL of H3K36me3 antibody (abcam; ab9050) or 5 μL of 8WG16 antibody (abcam; ab817). Immunoprecipitated DNA was quantified using real-time PCR (ABI; 7500). The primer sequences used in this study are listed as follows: C-box (5-GTCAAGCTCGTACCCACATC-3 and 5-CCGAAAGTATCTTGAGCCTCC-3), PLRE (5-CGGACGACGGCTGGCCAATTAG-3 and 5-TCGTGCTC TCTTGCTCACTTTCC-3), TSS (5- GAGGAACCAGAACGTAGCAG-3 and 5-GCAGGATAAACGGAGAAATGAC-3), ORF 5′ (5-TTACTTCATCTTCCGCACTGG-3 and 5-GGCAGGGTTACGATTGGATT-3), ORF Middle (5-GGACACCTTTCATTACAAACCG-3 and 5-TCCGCTAAAATCCCACTTCG-3), ORF 3′ (5-GATACCGAGACTGATGTGCG-3 and 5-AGCATGTCCACCTCTTTTCC-3), 3′ UTR (5-GAGAGCAAAAGGAACGCATTG-3 and 5-CTCCCCTGAAAATGGCAAAG-3). ChIP-qCR data were normalized to the sample of input DNA and presented as percentage of input DNA. Each experiment was carried out 3 times independently.

### Quantifications and statistical analyses

The Quantity one software was used to do the quantification for the Western Blot and Northern Blot. Error bars are standard deviations for race tube assays, ChIP assays, Western Blot and Northern Blot. All experiments were performed at least three independent times. Statistical significance was carried out by Student’s *t-* test, Ordinary one-way ANOVA Multiple comparisons or Lognormal ordinary one-way ANOVA Multiple comparisons.

## Supporting information

S1 Fig PAF-1 is required for keeping the normal circadian period in *Neurospora crassa.* (A) Race tube assay showing the circadian conidiation peak and period length of WT, *paf-1*^*KO*^, *ctr-9*^*KO*^ and *rtf-1*^*KO*^ strains in LD cycle (Light for 12 hours and Dark for 12 hours). Error bars are means ± SD. (n = 3). (B) Race tube assay showing the circadian conidiation rhythms and period length of WT, *paf-1*^*KO*^, *paf-1*^*KO*^;qa-Myc-PAF-1, *ctr-9*^*KO*^, *ctr-9*^*KO*^;qa-Myc-CTR-9, *rtf-1*^*KO*^, *rtf-1*^*KO*^;qa-Myc-RTF-1 strains. The concentration of the quinic acid (QA) in race tube assay is 0 M (Upper) and 1 × 10^-3^ M (Lower). Error bars are means ± SD. (n = 3). (C-D) Luciferase reporter assay showing the *frq* promoter activity in the *wt*;*frq-luc* and *paf-1*^*KO*^;*frq-luc* strains grown in DD. Raw data are normalized to subtract the baseline calculated by the LumiCycle analysis software. The raw data is shown in (C). The normalized luciferase activity is shown in (D). Error bars are means ± SD. (n = 2). (E) Western blot analysis showing the degradation rate of FRQ in the WT, *paf-1*^*KO*^, *ctr-9*^*KO*^, *rtf-1*^*KO*^ strains after the addition of cycloheximide (CHX, 10 μg/mL). Cultures were first grown in LL for 1 day prior to the addition of CHX and harvested at the indicated time. Quantification of the FRQ protein levels is shown in Right. The PVDF membrane (mem) stained with Coomassie blue was used as a loading control. Error bars are means ± SD. (n = 3).
(DOCX)

S2 Fig PAF-1, CTR-9, and RTF-1 specifically bind at the *frq* locus. (A) Western blot analysis showing that the antiserum specifically recognizes the PAF-1, CTR-9 or RTF-1 protein in the wild-type strain but not in the *paf-1*^*KO*^, *ctr-9*^*KO*^, *rtf-1*^*KO*^ strains respectively. (B-D) ChIP data showing that PAF-1 (B), CTR-9 (C), and RTF-1 (D) specially localized to 3’ region of the *frq* ORF, but not to the negative control gene *NCU05093*. Error bars are means ± SD. (n = 3). Significance difference was assessed by Student’s *t*-test. ***P < 0.001.
(DOCX)

S3 Fig PAF-1, CTR-9, LEO-1 and CDC-73 form a complex in which RTF-1 is not included. (A-D) Co-IP assays showing Myc-tagged PAF-1 interacts with CTR-9 but not with RTF-1 (A); Myc-tagged LEO-1 interacts with CTR-9, PAF-1 but not with RTF-1 (B); Myc-tagged CDC-73 interacts with CTR-9, PAF-1 but not with RTF-1 (C); Myc-tagged RTF-1 binds to neither PAF-1 nor CTR-9 (D).
(DOCX)

S4 Fig RAD-6 and BRE-1 are required for keeping the normal circadian period in *Neurospora crassa.* (A) Race tube assay showing the circadian conidiation peak and period length of WT, *rad-6*^*KO*^, *bre-1*^*KO*^ strains in LD cycle (Light for 12 hours and Dark for 12 hours). Error bars are means ± SD. (n = 3). (B) Race tube assay showing the circadian conidiation rhythms and period length of WT, *rad-6*^*KO*^, *bre-1*^*KO*^, *rad-6*^*KO*^;qa-Flag-RAD-6 and *bre-1*^*KO*^;qa-Myc-BRE-1 strains. The concentration of the quinic acid (QA) in race tube assay is 0 M (upper) and 1 × 10^-3^ M (lower). Error bars are means ± SD. (n = 3). (C) The amino acid alignment showing the ubiquitylation site of histone H2B is highly conserved among *Neurospora crassa* (Nc), *Saccharomyces cerevisiae* (Sc) and *Homo sapiens* (Hs).
(DOCX)

S5 Fig Ectopic overexpression of RAD-6 or BRE-1 fails to rescue the shortened conidiation period of *paf-1*^*KO*^ strain. (A) Race tube assays showing the conidiation period of WT, *paf-1*^*KO*^, *paf-1*^*KO*^;qa-Myc-PAF-1, *paf-1*^*KO*^;qa-Flag-RAD-6, *paf-1*^*KO*^;qa-Myc-BRE-1 strains. The concentration of the quinic acid (QA) in race assay is 1 × 10^-3^M. Error bars are means ± SD. (n = 3). (B) Co-IP assay showing RTF-1 and BRE-1 interact with each other.
(DOCX)

S6 Fig RTF-1 subunit is highly conserved among different species. (A) Sequence alignment of the RTF-1 protein among the *Neurospora crassa*, *Saccharomyces cerevisiae*, *Drosophila melanogaster* and *Homo sapiens*. (B) Western blot analysis showing that the expression levels of endogenous and Myc-tagged RTF-1 and its variants in WT, *rtf-1*^*KO*^, *rtf-1*^*KO*^;qa-Myc-RTF-1, *rtf-1*^*KO*^;qa-Myc-RTF-1^△3-23^, *rtf-1*^*KO*^;qa-Myc-RTF-1^E132K^, *rtf-1*^*KO*^;qa-Myc-RTF-1^130-132A^, *rtf-1*^*KO*^;qa-Myc-RTF-1^△221-263^, *rtf-1*^*KO*^;qa-Myc-RTF-1^△526-601^ strains. The membrane (mem) stained with Coomassie blue was used as a loading control.
(DOCX)

S1 Data Source data file. (XLSX)
