# Supplementary figures and images for: Paf1C regulates the Neurospora circadian clock by promoting the transcription elongation of frequency
Source: PLoS Genet. 2025 Oct 23;21(10):e1011926. doi: 10.1371/journal.pgen.1011926 (PMC12611138; doi:10.1371/journal.pgen.1011926)

**S1 Fig. PAF-1 is required for keeping the normal circadian period in *Neurospora crassa*.**


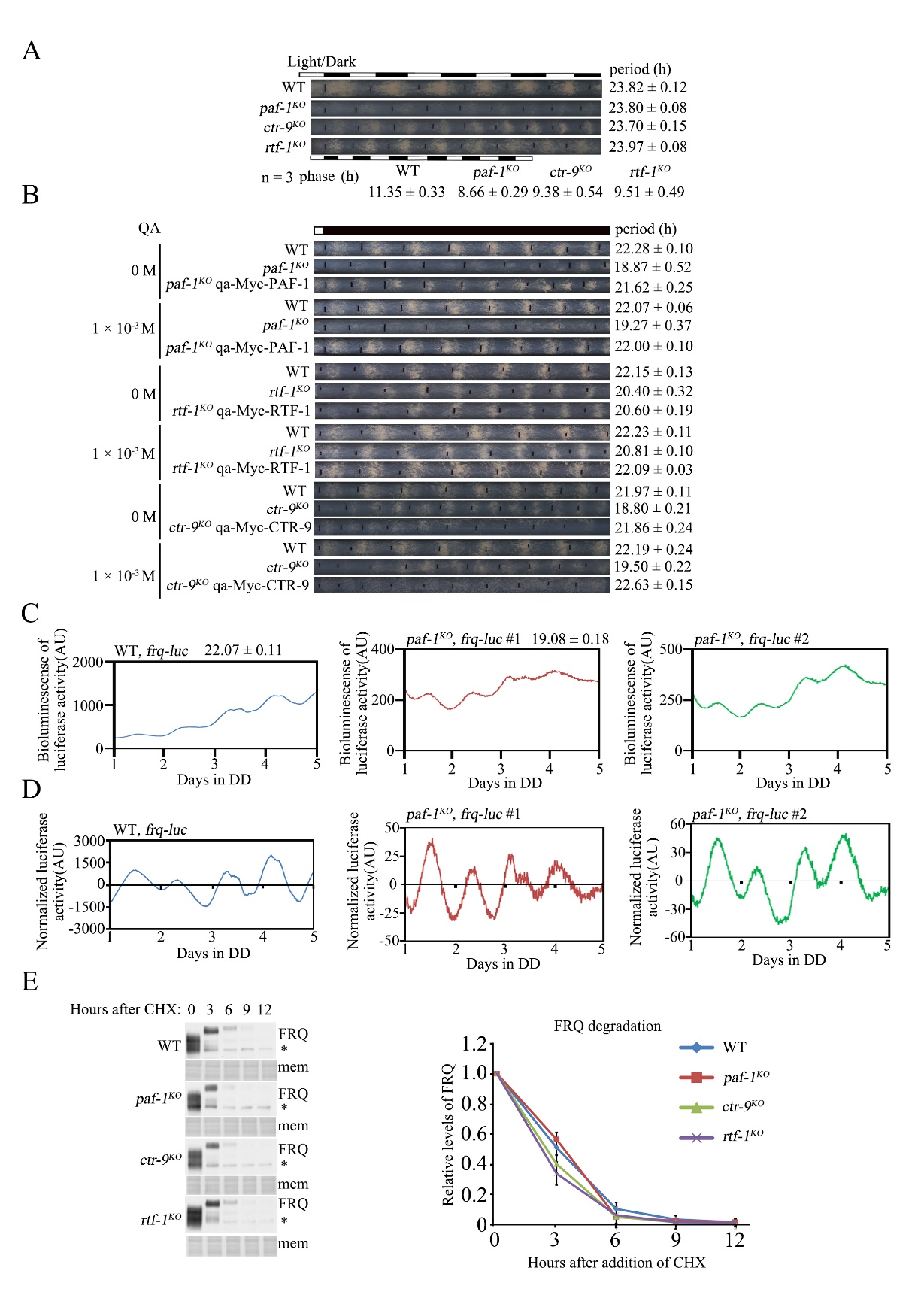

Supplement: S1 Fig — (A) Race tube assay showing the circadian conidiation peak and period length of WT, paf-1KO, ctr-9KO and rtf-1KO strains in LD cycle (Light for 12 hours and Dark for 12 hours). Error bars are means ± SD. (n = 3). (B) Race tube assay showing the circadian conidiation rhythms and period length of WT, paf-1KO, paf-1KO;qa-Myc-PAF-1, ctr-9KO, ctr-9KO;qa-Myc-CTR-9, rtf-1KO, rtf-1KO;qa-Myc-RTF-1 strains. The concentration of the quinic acid (QA) in race tube assay is 0 M (Upper) and 1 × 10^-3^ M (Lower). Error bars are means ± SD. (n = 3). (C-D) Luciferase reporter assay showing the frq promoter activity in the wt;frq-luc and paf-1KO;frq-luc strains grown in DD. Raw data are normalized to subtract the baseline calculated by the LumiCycle analysis software. The raw data is shown in (C). The normalized luciferase activity is shown in (D). Error bars are means ± SD. (n = 2). (E) Western blot analysis showing the degradation rate of FRQ in the WT, paf-1KO, ctr-9KO, rtf-1KO strains after the addition of cycloheximide (CHX, 10 μg/mL). Cultures were first grown in LL for 1 day prior to the addition of CHX and harvested at the indicated time. Quantification of the FRQ protein levels is shown in Right. The PVDF membrane (mem) stained with Coomassie blue was used as a loading control. Error bars are means ± SD. (n = 3). (DOCX) [file pgen.1011926.s001.docx]

**S2 Fig.** **PAF-1, CTR-9, and RTF-1 specifically bind at the *frq* locus.**


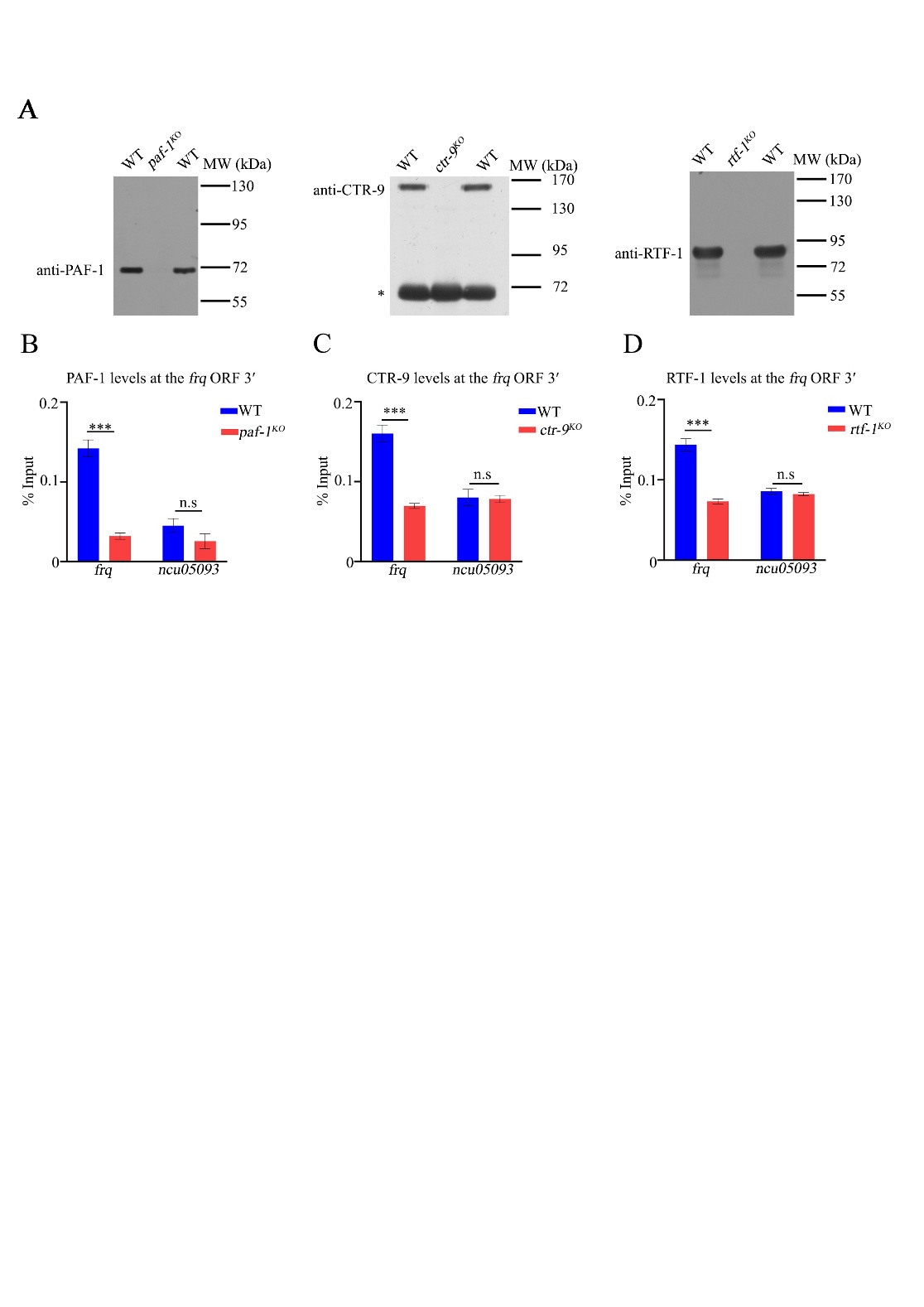

Supplement: S2 Fig — (A) Western blot analysis showing that the antiserum specifically recognizes the PAF-1, CTR-9 or RTF-1 protein in the wild-type strain but not in the paf-1KO, ctr-9KO, rtf-1KO strains respectively. (B-D) ChIP data showing that PAF-1 (B), CTR-9 (C), and RTF-1 (D) specially localized to 3’ region of the frq ORF, but not to the negative control gene NCU05093. Error bars are means ± SD. (n = 3). Significance difference was assessed by Student’s t-test. ***P < 0.001. (DOCX) [file pgen.1011926.s002.docx]

**S3 Fig.** **PAF-1, CTR-9, LEO-1 and CDC-73 form a complex in which RTF-1 is not included.**


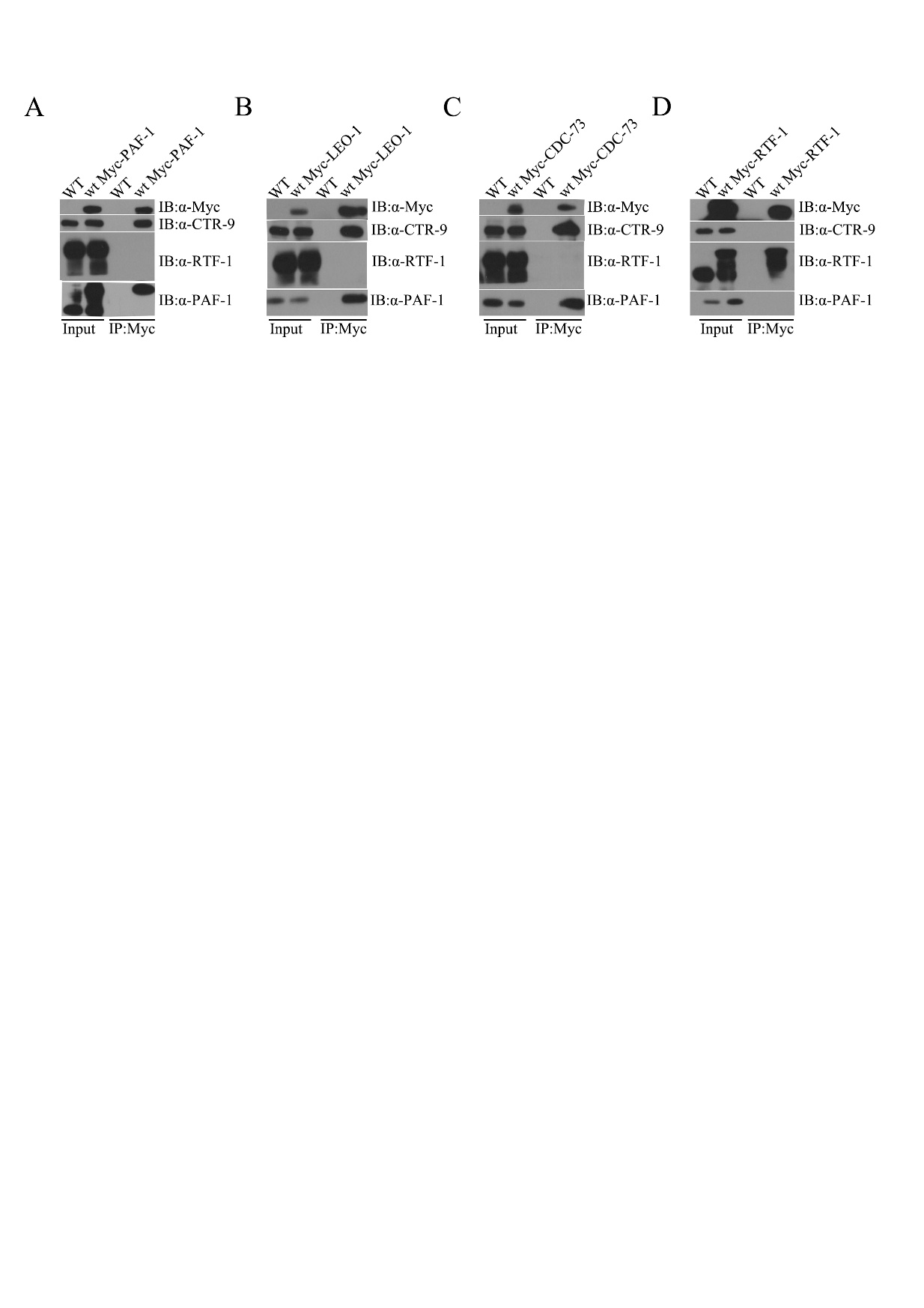

Supplement: S3 Fig — (A-D) Co-IP assays showing Myc-tagged PAF-1 interacts with CTR-9 but not with RTF-1 (A); Myc-tagged LEO-1 interacts with CTR-9, PAF-1 but not with RTF-1 (B); Myc-tagged CDC-73 interacts with CTR-9, PAF-1 but not with RTF-1 (C); Myc-tagged RTF-1 binds to neither PAF-1 nor CTR-9 (D). (DOCX) [file pgen.1011926.s003.docx]

**S4 Fig. RAD-6 and BRE-1 are required for keeping the normal circadian period in *Neurospora crassa*.**


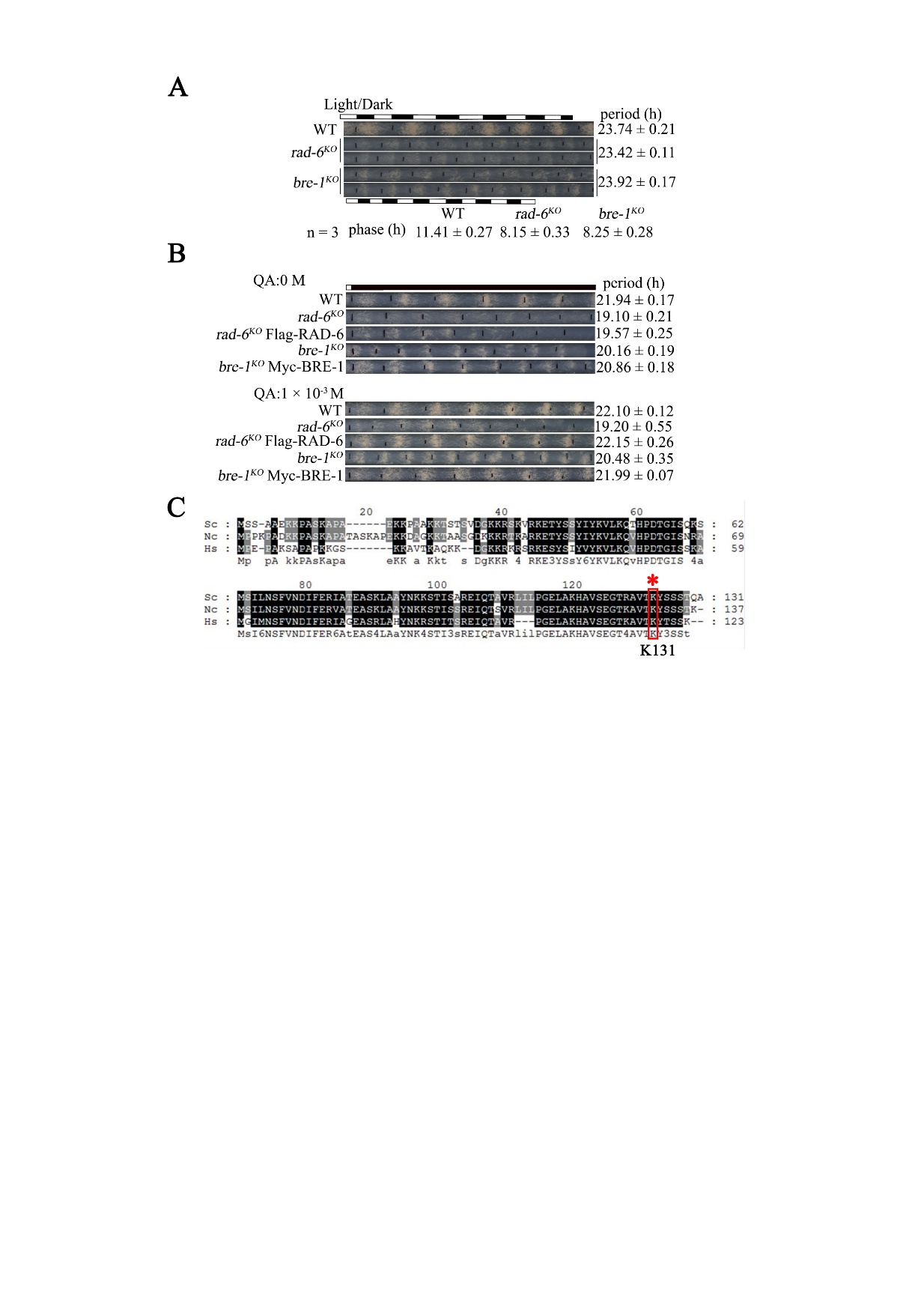

Supplement: S4 Fig — (A) Race tube assay showing the circadian conidiation peak and period length of WT, rad-6KO, bre-1KO strains in LD cycle (Light for 12 hours and Dark for 12 hours). Error bars are means ± SD. (n = 3). (B) Race tube assay showing the circadian conidiation rhythms and period length of WT, rad-6KO, bre-1KO, rad-6KO;qa-Flag-RAD-6 and bre-1KO;qa-Myc-BRE-1 strains. The concentration of the quinic acid (QA) in race tube assay is 0 M (upper) and 1 × 10^-3^ M (lower). Error bars are means ± SD. (n = 3). (C) The amino acid alignment showing the ubiquitylation site of histone H2B is highly conserved among Neurospora crassa (Nc), Saccharomyces cerevisiae (Sc) and Homo sapiens (Hs). (DOCX) [file pgen.1011926.s004.docx]

**S6 Fig.** **RTF-1 subunit is highly conserved among different species.**


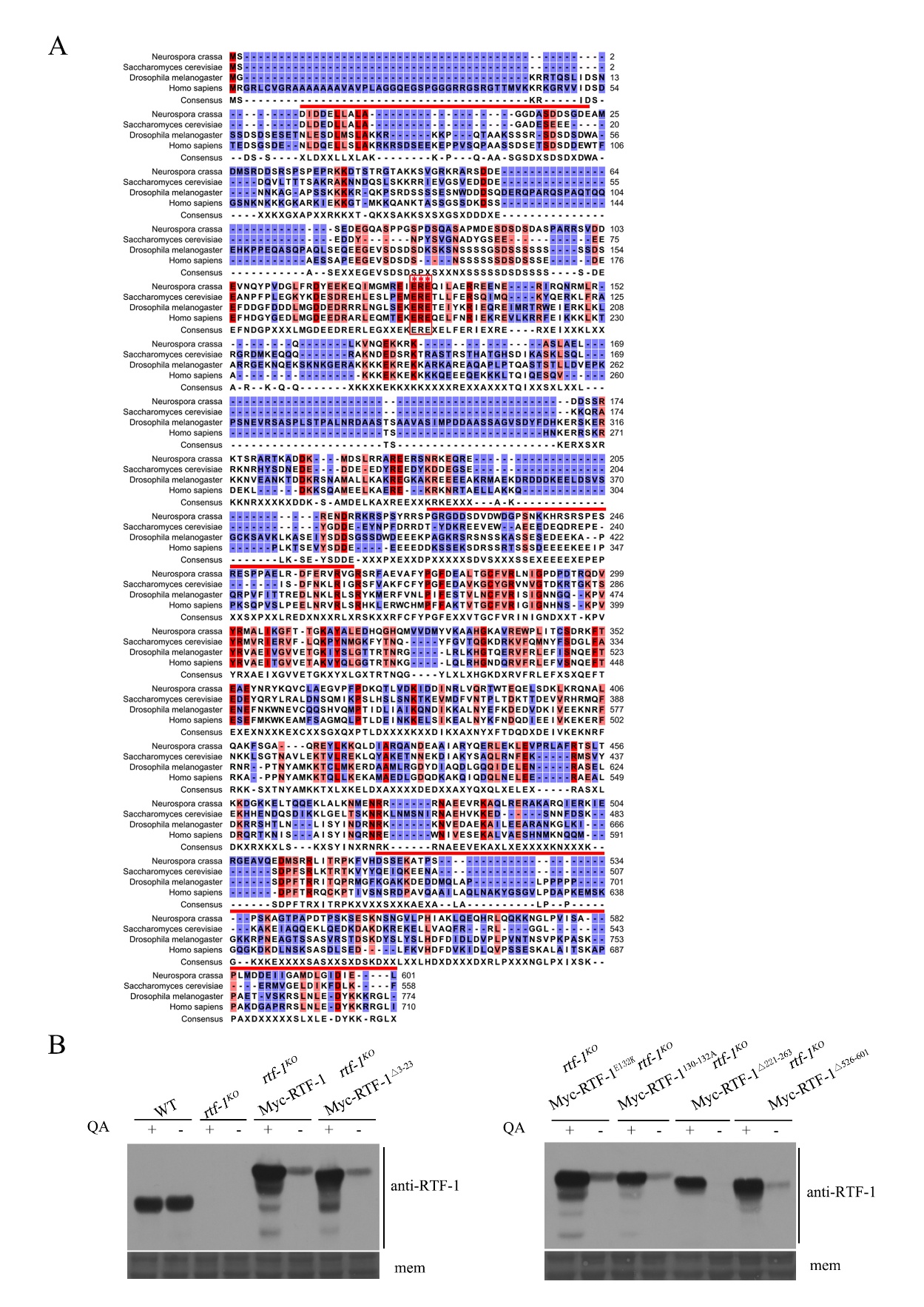

Supplement: S6 Fig — (A) Sequence alignment of the RTF-1 protein among the Neurospora crassa, Saccharomyces cerevisiae, Drosophila melanogaster and Homo sapiens. (B) Western blot analysis showing that the expression levels of endogenous and Myc-tagged RTF-1 and its variants in WT, rtf-1KO, rtf-1KO;qa-Myc-RTF-1, rtf-1KO;qa-Myc-RTF-1△3-23, rtf-1KO;qa-Myc-RTF-1E132K, rtf-1KO;qa-Myc-RTF-1130-132A, rtf-1KO;qa-Myc-RTF-1△221-263, rtf-1KO;qa-Myc-RTF-1△526-601 strains. The membrane (mem) stained with Coomassie blue was used as a loading control. (DOCX) [file pgen.1011926.s006.docx]
