# Supplementary material for: Paf1C regulates the Neurospora circadian clock by promoting the transcription elongation of frequency
Source: PLoS Genet. 2025 Oct 23;21(10):e1011926. doi: 10.1371/journal.pgen.1011926 (PMC12611138; doi:10.1371/journal.pgen.1011926)

**S5 Fig.** **Ectopic overexpression of RAD-6 or BRE-1 fails to rescue the shortened conidiation period of *paf-1^KO^* strain.**


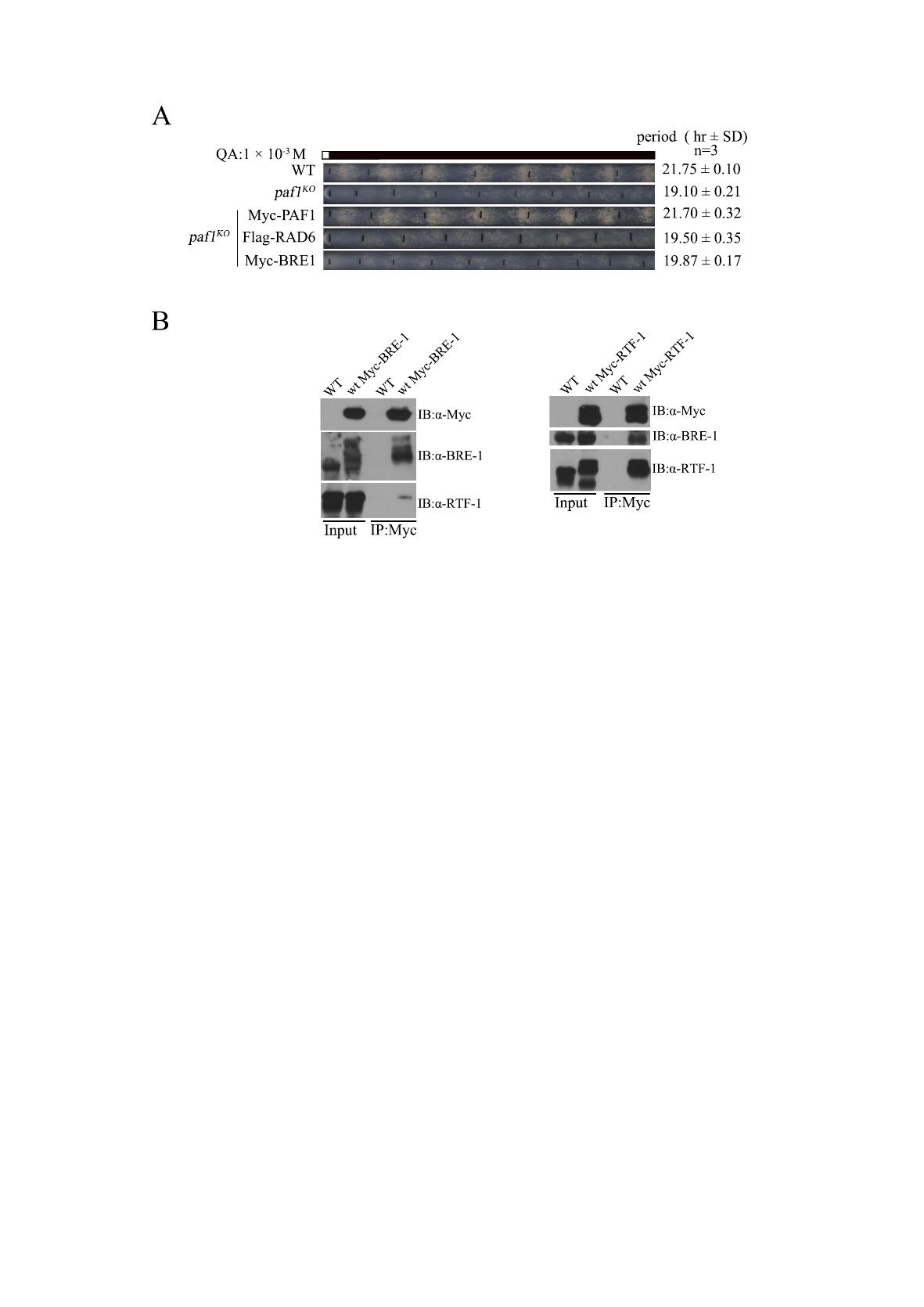

Supplement: S5 Fig — (A) Race tube assays showing the conidiation period of WT, paf-1KO, paf-1KO;qa-Myc-PAF-1, paf-1KO;qa-Flag-RAD-6, paf-1KO;qa-Myc-BRE-1 strains. The concentration of the quinic acid (QA) in race assay is 1 × 10^-3^M. Error bars are means ± SD. (n = 3). (B) Co-IP assay showing RTF-1 and BRE-1 interact with each other. (DOCX) [file pgen.1011926.s005.docx]
